# Influence of Ocean Acidification on a Natural Winter-to-Summer Plankton Succession: First Insights from a Long-Term Mesocosm Study Draw Attention to Periods of Low Nutrient Concentrations

**DOI:** 10.1371/journal.pone.0159068

**Published:** 2016-08-15

**Authors:** Lennart T. Bach, Jan Taucher, Tim Boxhammer, Andrea Ludwig, The Kristineberg KOSMOS Consortium, Eric P. Achterberg, María Algueró-Muñiz, Leif G. Anderson, Jessica Bellworthy, Jan Büdenbender, Jan Czerny, Ylva Ericson, Mario Esposito, Matthias Fischer, Mathias Haunost, Dana Hellemann, Henriette G. Horn, Thomas Hornick, Jana Meyer, Michael Sswat, Maren Zark, Ulf Riebesell

**Affiliations:** 1 GEOMAR Helmholtz Centre for Ocean Research Kiel, Kiel, Germany; 2 Alfred-Wegener-Institut Helmholtz-Zentrum für Polar- und Meeresforschung, Biologische Anstalt Helgoland, Helgoland, Germany; 3 Department of Marine Sciences, University of Gothenburg, Gothenburg, Sweden; 4 Ocean and Earth Sciences, University of Southampton, Southampton, United Kingdom; 5 The University Centre in Svalbard (UNIS), Longyearbyen, Norway; 6 Department of Environmental Sciences, University of Helsinki, Helsinki, Finland; 7 Leibniz Institute of Freshwater Ecology and Inland Fisheries (IGB), Experimental Limnology, Stechlin, Germany; 8 Institute for Chemistry and Biology of the Marine Environment (ICBM), Research Group for Marine Geochemistry (ICBM-MPI Bridging Group), Carl von Ossietzky University, Oldenburg, Germany; CSIR-National Institute of Oceanography, INDIA

## Abstract

Every year, the oceans absorb about 30% of anthropogenic carbon dioxide (CO_2_) leading to a re-equilibration of the marine carbonate system and decreasing seawater pH. Today, there is increasing awareness that these changes–summarized by the term ocean acidification (OA)–could differentially affect the competitive ability of marine organisms, thereby provoking a restructuring of marine ecosystems and biogeochemical element cycles. In winter 2013, we deployed ten pelagic mesocosms in the Gullmar Fjord at the Swedish west coast in order to study the effect of OA on plankton ecology and biogeochemistry under close to natural conditions. Five of the ten mesocosms were left unperturbed and served as controls (~380 μatm *p*CO_2_), whereas the others were enriched with CO_2_-saturated water to simulate realistic end-of-the-century carbonate chemistry conditions (~760 μatm *p*CO_2_). We ran the experiment for 113 days which allowed us to study the influence of high CO_2_ on an entire winter-to-summer plankton succession and to investigate the potential of some plankton organisms for evolutionary adaptation to OA in their natural environment. This paper is the first in a PLOS collection and provides a detailed overview on the experimental design, important events, and the key complexities of such a “long-term mesocosm” approach. Furthermore, we analyzed whether simulated end-of-the-century carbonate chemistry conditions could lead to a significant restructuring of the plankton community in the course of the succession. At the level of detail analyzed in this overview paper we found that CO_2_-induced differences in plankton community composition were non-detectable during most of the succession except for a period where a phytoplankton bloom was fueled by remineralized nutrients. These results indicate: (1) Long-term studies with pelagic ecosystems are necessary to uncover OA-sensitive stages of succession. (2) Plankton communities fueled by regenerated nutrients may be more responsive to changing carbonate chemistry than those having access to high inorganic nutrient concentrations and may deserve particular attention in future studies.

## 1 Introduction

The oceans absorb currently about 2 gigatons carbon as anthropogenic CO_2_ per year [1]. In seawater, most of the anthropogenic CO_2_ reacts with H_2_O to form carbonic acid. The subsequent dissociation of carbonic acid causes a prominent decline in the seawater pH and major shifts in the marine carbonate system–a process called “ocean acidification” [2,3]. Studies investigating the consequences of ocean acidification (OA) for marine life have primarily focused on physiological processes of individual organisms and the experiments were usually conducted with OA-acclimated rather than OA-adapted individuals [4]. However, OA takes place in natural ecosystems with complex species interactions and occurs on timescales long enough to provide the opportunity for evolutionary adaptation [5,6]. Hence, our understanding of OA effects on marine biota must advance from a single species to a whole ecosystem level and our experimental design should ideally consider timescales which cover the entire plankton succession and are long enough to include evolutionary adaptation [7].

So far, OA studies comprising entire ecosystems were primarily focused on benthic habitats near volcanic CO_2_ vent sites [8–11] whereas fewer studies have been made in pelagic ecosystems which are more difficult to study due to local displacement of the plankton community [12]. Therefore, much of our understanding on the impacts of OA on plankton communities derives from (short-term) incubation experiments with relatively small volume [13]. These experiments are particularly valuable when aiming to investigate physiological and ecological changes on the lowest trophic levels. However, the duration of such experiments is limited due to technical restrictions with small incubation volumes and they are therefore in most cases inadequate to study an entire plankton succession. Furthermore, they are limited when aiming to unravel the potential consequences of OA-induced changes in the plankton community on key biogeochemical traits such as organic matter export as these investigations require large sample volumes generated by plankton communities representative of the study site. *In-situ* mesocosm experiments with large incubation volumes are one option to bridge this gap as plankton communities can be sustained for long enough time to study the seasonal succession of natural plankton communities in their natural habitat [14] without too much bias towards smaller functional groups [15]. However, *in-situ* long-term studies with large incubation volumes are technically, logistically, and financially challenging and thus require strong institutional support and a well-coordinated collaborative effort of many scientists and technicians. From January to July 2013 we faced this challenge and conducted the “BIOACID II long-term mesocosm study” hosted by the Sven Lovén Centre for Marine Sciences, Kristineberg located on the Skagerrak coast (west coast of Sweden). In total, 55 scientists and technicians from 11 different institutes participated actively in this study with the aim to investigate the impact of OA on physiological, ecological, evolutionary [16], and biogeochemical processes in a natural winter-to-summer plankton succession.

The present paper is the first within this PLOS collection and has two primary intentions. First, we aim to provide a detailed overview on the study site, starting conditions, background data, and key events during the study, thereby setting the scene for the more specialized papers published within the framework of this mesocosm experiment (a summary of intended publications is provided in S1 Table). Second, we will investigate on a relatively coarse functional/taxonomic resolution (in this overview paper) whether realistic end of the century carbonate chemistry conditions (i.e. *p*CO_2_ = 760 μatm; [17]) can restructure plankton community composition over the course of a natural winter-to-summer plankton succession. This will help to uncover the critical phases where CO_2_ is particularly influential.

## 2 Materials and Methods

In this study we added herring eggs (*Clupea harengus*) to the mesocosms. Animal welfare was assured according to the ethical permission (number 332–2012), where it is stated that the species used is not endangered and that sacrificed specimens were anaesthetized beforehand with MS-222, so stress was reduced to a minimum.

### 2.1 The study site

**Fig 1:**
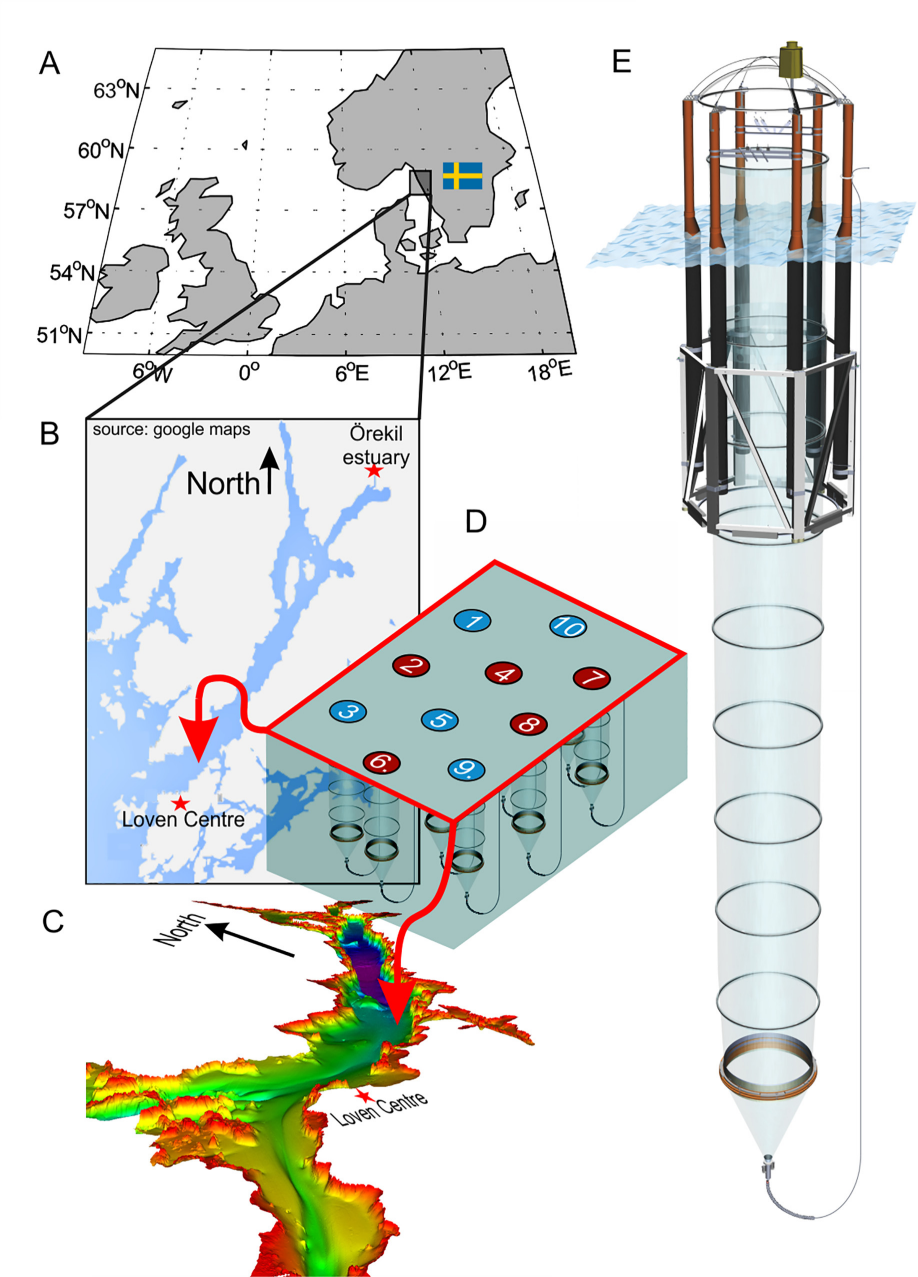
Study site and mesocosm deployment. (A) Map of north-western Europe. The small black square marks the study site off the Swedish west coast. (B) Close-up on the Gullmar Fjord region. (C) Bathymetric map of Gullmar Fjord [19]. The mesocosm deployment site was on the inner edge of the sill, close to the fjord mouth (marked on (B) and (C) by the red arrows). (D) Arrangement of the 10 mesocosms at deployment site (see Table 2 for coordinates). Small numbers inside the circles show mesocosm arrangement (M1-M10) whereas blue and red represent ambient and high CO_2_ replicates, respectively. (E) Schematic drawing of a mesocosm unit. The floatation frame is 8 m high. The bag without sediment trap extends 17 m below sea surface and has a diameter of 2 m. The sediment trap is attached to the bag with a flange ring and reaches down to 19 m water depth.

**Fig 2:**
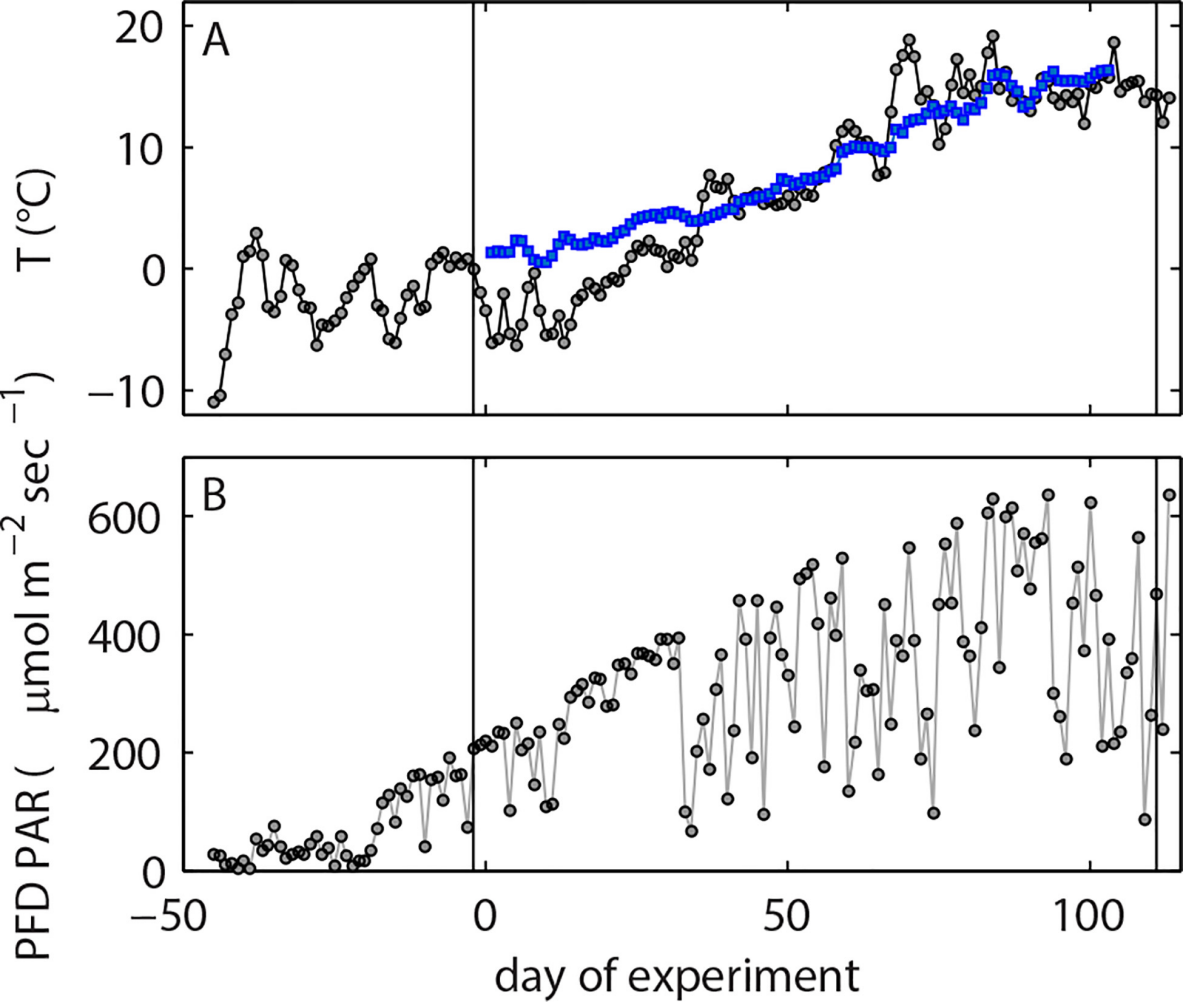
Daily averaged (A) air temperature (grey), surface water temperature (blue) and (B) photon flux density (PFD) of photosynthetic active radiation (PAR). Air temperature and PAR data were recorded on the roof of the Sven Lovén Centre (~3 km distance from the mesocosm deployment site) and downloaded from http://www.weather.loven.gu.se/kristineberg/data.shtml. Surface water temperature was recorded with HOBO pro v2 data logger mounted at 0.1 m depth in M2. Note that temperature development was quasi identical in all mesocosms (S3 Fig). The timeline starts with the arrival of research vessel *Alkor* at Gullmar Fjord on the 23^rd^ of January, 2013 (t-45). Grey vertical lines indicate the start (7^th^ of March; t-2) and the end (28^th^ of June; t111) of the successful experiment.

The Gullmar Fjord is located approximately 100 km north of Gothenburg on the Swedish west coast (Fig 1A). It extends 28 km inland in a north-easterly direction and is about 1–2 km wide (Fig 1B). It was shaped by a seaward moving glacier which formed the 116 m deep inner basin and the shallower sill (43 m) at the entrance of the fjord (Fig 1C; [18,19]). Water below sill level in the inner basin is filled with relatively saline (S>33) North Sea and/or North Atlantic water which has a prolonged residence time of about one year due to entrapment by the sill barrier ([20]; Fig 1C). The exchange of water above sill level is considerably faster (16–40 days; [21]). It is primarily driven by wind stress since tidal forcing is usually below 0.2 m in this region [18]. The water column above sill level is composed of three major water bodies: (1) a thin low-salinity top layer (usually less than 1 m) primarily due to freshwater discharge from the Örekil River located at the landward end of the fjord (Fig 1B); (2) a brackish seawater (S<30) layer fed by the northward moving Baltic current which transports low salinity water from the Baltic proper along the Swedish coast through the Kattegat where it gradually mixes with North Sea water; (3) a marine (S>30) layer fed by North Atlantic and/or North Sea water from the Skagerrak which constitutes the majority of the water on top of the sill and the entrapped basin water. The halocline, separating brackish surface water from underlying marine water, is usually between 5–20 m [18,21,22].

### 2.2 Mesocosm deployment and initiation of the experiment

On the 29^th^ of January 2013, ten “Kiel Off-Shore Mesocosms for Future Ocean Simulations” (KOSMOS, M1-M10; [23]) were deployed by research vessel *Alkor* close to the fjord entrance at the inner edge of the sill (58° 15.981’ N, 11° 28.699’ E; Fig 1) at a water depth of ~60–80 m (Fig 1, Table 1, Table 2). The cylindrical but initially folded mesocosm bags (2 m diameter) made of thermoplastic polyurethane foil were mounted in 8 m high flotation frames (Fig 1E). The bags were unfolded immediately after deployment in such a way that the lower opening of the bags reached a depth of 19 m, while the upper opening was positioned 1 m below surface. Both the upper and lower openings were covered with meshes (3 mm mesh size) in order to exclude patchily distributed nekton and large zooplankton like fish larvae or jelly fish from the enclosed water body. On the 12^th^ of February, divers replaced the meshes at the bottom of mesocosm bags with 2 m long conical sediment traps thereby sealing the bottom of the mesocosms. Simultaneously, the boat crew pulled the upper part of the bags above the sea surface so that the water body within mesocosms was isolated from this time onwards (Fig 1E) and the experiment started (Table 1). Mesocosm closing lasted for less than 1 hour in total, thereby minimizing differences between the enclosed water in each mesocosm.

**Table 1:** Sampling and maintenance schedule during the mesocosm study.

Date	t day	STS	WCS	CTD	net haul	CLEAN	seed	Event
23/01/2013	t-45							Arrival of RV Alkor on study site
29/01/2013	t-39							Deployment of all 10 mesocosms in Gullmar Fjord (exact location shown in Fig 1)
12/02/2013	t-25							Closing all 10 mesocosms (i.e. start of the failed experiment)
03/03/2013	t-6							Opening all 10 mesocosms (i.e. end of the failed experiment)
06/03/2013	t-3							Cleaning of mesocosm bags and servicing of sediment traps on shore
07/03/2013	t-2							Closing all 10 mesocosms (i.e. start of the successful experiment), mixing water column (5 minutes), hole detected in M1
08/03/2013	t-1				1			1^st^ CO_2_ enrichment
09/03/2013	t0							Mixing water column (4 minutes), 2^nd^ CO_2_ enrichment, N_2_O tracer addition (M3, M5, M7, M8)
10/03/2013	t1				2			Hole detected in M8
11/03/2013	t2							Sampling (15 L) for nutrients and microzooplankton grazing incubations, 3^rd^ CO_2_ enrichment, mixing M8 for 5 minutes with subsequent CTD cast to spot the hole, hole detected in M1
12/03/2013	t3				1			Diving with rebreather inside M1 and M8 and fixing holes of both mesocosms from the outside.
13/03/2013	t4							4^th^ CO_2_ enrichment
14/03/2013	t5							
15/03/2013	t6							While cleaning a 1 mm mesh was attached to the cleaning ring to remove fish larvae and jelly fish, outside cleaning (0–1.5 m)
16/03/2013	t7							
17/03/2013	t8							
18/03/2013	t9				1			
19/03/2013	t10							
20/03/2013	t11				1			
21/03/2013	t12							Deployment of benthos and biofilm plates in all mesocosms, diving with rebreather inside M6 to recover lost device
22/03/2013	t13							
23/03/2013	t14							Outside cleaning (1.5–8 m)
24/03/2013	t15							
25/03/2013	t16							
26/03/2013	t17				2			5^th^ CO_2_ enrichment
27/03/2013	t18							Outside cleaning (0–1.5 m), sampling for microzooplankton grazing experiments
28/03/2013	t19				2			
29/03/2013	t20							
30/03/2013	t21							
31/03/2013	t22							
01/04/2013	t23							
02/04/2013	t24							
03/04/2013	t25				3			
04/04/2013	t26							Biofilm sampling
05/04/2013	t27				1			
06/04/2013	t28							Outside cleaning (3–5 m)
07/04/2013	t29							
08/04/2013	t30							
09/04/2013	t31							Installation of light and temperature loggers in M4 and M10
10/04/2013	t32							Sampling of 40 L for light stress experiments, recovery of light and temperature loggers
11/04/2013	t33				3			
12/04/2013	t34							Sampling for microzooplankton grazing incubations (15 L)
13/04/2013	t35							Sediment trap collector of M5 was opened for ~1 min to recover a lost device
14/04/2013	t36							
15/04/2013	t37							Establishment of thermal stratification (Fig 5)
16/04/2013	t38							N_2_O tracer addition to M3, M5, M7, and M8
17/04/2013	t39				1			
18/04/2013	t40							
19/04/2013	t41							Sediment trap collector of M2 was opened for ~1 min to remove clogging
20/04/2013	t42							
21/04/2013	t43							Biofilm sampling
22/04/2013	t44							Outside cleaning (6–8 m)
23/04/2013	t45							
24/04/2013	t46							6^th^ CO_2_ enrichment, 1^st^ brine (NaCL) addition to all mesocosms for volume determination, hole detected in M2
25/04/2013	t47							Hole fixed in M2
26/04/2013	t48							7^th^ CO_2_ enrichment, addition of herring egg incubators at 3 m depth
27/04/2013	t49				2			
28/04/2013	t50							
29/04/2013	t51							
30/04/2013	t52							
01/05/2013	t53							Lowering herring egg incubators from 3 to 6 m depth, hole detected in M9
02/05/2013	t54							
03/05/2013	t55							
04/05/2013	t56							Addition of sea urchin larvae, biofilm sampling
05/05/2013	t57				2			
06/05/2013	t58							Hole fixed in M9
07/05/2013	t59				2			
08/05/2013	t60							Outside cleaning (5–7 m)
09/05/2013	t61							
10/05/2013	t62							Biofilm sampling
11/05/2013	t63							Peak hatch of herring larvae
12/05/2013	t64							Recovery of herring egg incubators
13/05/2013	t65				2			
14/05/2013	t66							Diving with rebreather in M9, deployment of temperature and light loggers in M4 and M10
15/05/2013	t67							
16/05/2013	t68							Biofilm sampling, 8^th^ CO_2_ enrichment
17/05/2013	t69							
18/05/2013	t70							
19/05/2013	t71							
20/05/2013	t72							Outside cleaning (0–1.5 m)
21/05/2013	t73				2			
22/05/2013	t74							
23/05/2013	t75							
24/05/2013	t76				1			Outside cleaning (15–17 m)
25/05/2013	t77							
26/05/2013	t78							Biofilm sampling
27/05/2013	t79							
28/05/2013	t80							Outside cleaning (3–5 m)
29/05/2013	t81				2			Biofilm sampling
30/05/2013	t82							Outside cleaning (5–10 m)
31/05/2013	t83							
01/06/2013	t84							Outside cleaning (10–12 m)
02/06/2013	t85							
03/06/2013	t86							Outside cleaning (12–14 m)
04/06/2013	t87							
05/06/2013	t88							9^th^ CO_2_ enrichment
06/06/2013	t89				2			
07/06/2013	t90							
08/06/2013	t91							
09/06/2013	t92							
10/06/2013	t93							
11/06/2013	t94							
12/06/2013	t95							
13/06/2013	t96				2			Net hauls with a 10 μm net, biofilm sampling
14/06/2013	t97				2			
15/06/2013	t98							Outside cleaning (8–10 m)
16/06/2013	t99							
17/06/2013	t100							
18/06/2013	t101							
19/06/2013	t102							Cleaning inner part of the sediment traps from the outside with magnetic brushes
20/06/2013	t103				1		μ	One net haul with 10 μm net
21/06/2013	t104							A 1 mm mesh was attached to the cleaning ring to recover herring larvae
22/06/2013	t105							Hole detected in M8 and M9
23/06/2013	t106							Hole fixed in M8 but not in M9
24/06/2013	t107				2			
25/06/2013	t108				8			All net hauls with 10 μm net
26/06/2013	t109							
27/06/2013	t110							Biofilm sampling
28/06/2013	t111							End of the experiment

Days of experiment (t days) relate to the day where the water column was fully homogeneous after mixing (t0). Filled grey areas are events of: Sediment trap sampling (STS), water column sampling (WCS), CTD casts, net hauls (with the number indicating how many net hauls were done), cleaning the inside of the mesocosm bags (CLEAN), and water column seeding (seed).

**Table 2:** Overview of mesocosm setup.

	mooring position		Enclosed water mass		Mean *p*CO_2_ (μatm)				
Mesocosm	North	East	weight (ton)	estimated influx through holes (% of total volume)	phase I	phase II	phase III	phase IV	total
M1	58° 16.008'	11° 28.680‘	51.3	2.4 (t-1—t3)	370	318	341	425	365
M2	58° 15.995‘	11° 28.659‘	55.9	2.8 (t39—t45)	745	629	759	864	759
M3	58° 15.983‘	11° 28.639‘	47.5		365	342	385	472	398
M4	58° 15.981	11° 28.699‘	51.6		754	615	719	865	744
M5	58° 15.969‘	11° 28.678‘	47.9		366	346	393	481	404
M6	58° 15.955‘	11° 28.660‘	51.4		765	640	731	857	753
M7	58° 15.972‘	11° 28.767‘	49.1		779	637	745	876	765
M8	58° 15.961‘	11° 28.745‘	53.1	3 (t0—t5), 0.2 (t105)	765	686	754	865	773
M9	58° 15.949‘	11° 28.727‘	50.0	1.9 (t53—t63), 0.3 (t105—t111)	361	321	374	471	389
M10	58° 15.993’	11° 28.720‘	49.6		367	316	335	423	362

The volume of water enclosed in each mesocosm was determined on t46 of the experiment. *p*CO_2_ values are averages of the four phases and means over the entire study (total).

On the 3^rd^ of March we had to stop the experiment and recover the sediment traps due to technical problems (see section 3.1.1 for reasons and 3.2.1 for biological consequences). Therefore, mesocosm bags were lowered below surface to allow water exchange with the fjord. After repairing the sediment traps they were re-installed and all mesocosms were closed again on the 7^th^ of March as described above but without the use of the 3 mm meshes. Instead, a mesh with 1 mm mesh size was attached to the cleaning ring on day 6 (Table 1; cleaning ring application described in section 2.4) and passed through the mesocosms to remove large and often patchily distributed zooplankton and nekton. Very few organisms were caught, however, in this operation. The 7^th^ of March marks the beginning of the second experiment, which lasted for 113 days from t-2 until t111 (Table 1).

### 2.3 Mesocosm CO_2_ manipulations and salt additions

Five of the ten mesocosms (M1, M3, M5, M9, M10) were untreated controls while the other five (M2, M4, M6, M7, M8) were manipulated by adding CO_2_-saturated seawater [23]. In this manipulation technique, a filtered (20 μm) Gullmar Fjord surface water volume of about 1500 L is aerated with pure CO_2_ gas for about 1 hour to reach pH_NBS_ ~4 and subsequently transferred into smaller bottles of ~25 L which are closed airtight, without headspace, to avoid degassing. These bottles were transported to the mesocosms by boat where the aerated water was pumped into the high CO_2_ mesocoms through a distribution device which we call “the spider” as it has multiple small tubes which disperse the volume evenly within a radius of ~1 m. By pulling the spider up and down within each mesocosm, we ensured homogenous CO_2_ enrichment throughout the entire water column. Target *p*CO_2_ was reached initially by CO_2_ additions on four consecutive days with the first one being on the 8^th^ of March (t-1). Further CO_2_ additions in the course of the experiment were made on a regular basis to account for CO_2_ loss through outgassing (Table 1).

Adding precise known amounts of saturated NaCl brine to the mesocosms can be used to determine their volume as it is proportional to the measurable change in salinity [24]. Saturated NaCl brine was generated by dissolving 300 kg of NaCl in 1000 L of filtered (20 μm) Gullmar Fjord surface water. The brine was subsequently filled into 25 L bottles and evenly dispersed in the mesocosms on the 24^th^ of April (t46) with the spider as described above. For a detailed description of the procedure please refer to Czerny et al. [24].

### 2.4 Mesocosm cleaning

The mesocosm bags had to be cleaned from the in- and outside on a regular basis to avoid growth of a benthic community on the bags, which consume nutrients and reduce photon flux density inside the mesocosms. The outside of the bags was cleaned on a regular basis (Table 1) with brushes, either by boat crews (0–1 m depth) or by divers (1–19 m). The inside was cleaned down to the last segment of the bag with a cleaning ring specifically designed for this purpose [23]. Inside cleaning of the bags was conducted approximately every eighth day (Table 1) to disturb fouling by benthic organisms in very early stage of their succession. The inner side of the lowest segment and of the sediment trap was only cleaned at the very end of the experiment (t102). Note, however, that fouling by algae and epiphytes is reduced at this depth as the photon flux density reaching the sediment trap is fairly low and most large heterotrophs like mussels or barnacles do not attach to the flexible bag material.

### 2.5 Addition of organisms

Some organisms, characteristic for a winter-to-summer succession in this region may not have been present in the water column by the time the mesocosms were closed. We accounted for this problem by adding fjord water to the mesocosms every fourth day (Table 1) allowing plankton organisms to enter the mesocosm community. Seeding water was collected at deployment site with a submersible pump which was lowered steadily to 19 m depth in about 5 minutes, thereby transferring about 300 L of seawater into a large container placed on the sampling boat. The collected seawater was subsequently stirred carefully so that all organisms were distributed homogenously within the 300 L batch. 22 L of the stirred seawater (i.e. ~0.44 ‰ of the total mesocosm volume) was then added to each mesocosm with a bucket which was lowered to the water surface inside the mesocosms and emptied carefully. In total, 550 L fjord water was added to each mesocosm on 25 occasions (Table 1) which sums up to ~1% of the mesocosms’ volume.

Next to smaller planktonic organisms, we also added herring (*Clupea harengus*) and green sea-urchin (*Strongylocentrotus droebachiensis*) larvae to each mesocosm. Both species were released in relatively low densities (~90 herring eggs and 110 sea urchin larvae per m^3^) to minimize potential top-down-effects. Herring eggs were stuck on plastic plates and mounted in the middle of the cylindrical bags at 3 m depth from day 48 until peak hatching on day 63. Herring larvae started feeding on (most likely) copepod-nauplii and ciliates after the yolk-sac stage at around day 71, switching to bigger prey with growing size. Larvae of the green sea urchin were grown in the laboratory following Dorey et al. [25] until reaching the swimming gastrula stage and then gently added to the mesocosms on day 56 with a bucket in the same way as the seeding water.

### 2.6 Sampling and CTD operations

Sinking detritus was collected in the sediment traps at the bottom of the mesocosms. To avoid resuspension of the material we emptied the sediment traps before water column sampling using a vacuum system connected to a tube which was attached to the collecting cups following Boxhammer et al. [26].

Water column samples were collected every second day at 9 a.m. (local time) with “integrating water samplers” (IWS, Hydrobios) that sample a total volume of 5 L from 0–17 m depth evenly through the water column. The number of IWS hauls per mesocosm varied between sampling days but generally ranged between 4 and 8. More volume was needed on days when incubation experiments were conducted on shore. The volume of 2–4 IWS hauls was pooled in 1–2 ten liter carboys per mesocosm onboard the sampling boat. This water was later used for particulate matter (PM) analysis, pigment analysis, flow cytometry, and phytoplankton/microzooplankton microscopy. All carboys were stored in the dark on the boats until sampling was finished (usually between 10 and 12 a.m. local time) and then transferred into a temperature-controlled room on shore (set to in-situ temperature) where subsamples were taken (section 2.7). The volume of the remaining IWS hauls was used for gas or easily contaminable samples, which were transferred into separate sampling bottles on the sampling boats. These sensitive samples were: dissolved inorganic carbon (DIC), pH, halocarbons, nitrous oxide (N_2_O), inorganic nutrients (nitrate (NO_3_^-^), nitrite (NO_2_^-^), dissolved silicate (Si(OH)_4_), ammonium (NH_4_^+^), phosphate (PO_4_^3-^)), dissolved organic carbon/nitrogen (DOC, DON), and water for all *in vitro* incubation experiments such as primary production (^14^C) or bacterial protein production assays.

Gas and incubation samples were carefully transferred from the IWS into gas tight sampling bottles with TYGON tubes placed at the bottom of the bottle. Sample bottles were then filled bottom to top avoiding air bubbles and allowing an overflow of twice the bottle volume before they were closed without headspace. All bottles were rinsed with sample water before the actual sample was taken. Inorganic nutrient samples were filled into 200 mL acid washed (10% HCl) PVC bottles. Samples for DOC and DON determination were transferred from IWS into pre-combusted (400°C, 4 h) glass vials (Whatman), after filtration through pre-combusted glass fibre filters (GF/F, nominal pore size 0.7 μm, Whatman). All samples tapped from the IWS on board were stored in boxes and in the shade until sampling was finished.

Zooplankton samples were collected around 3 p.m. (local time) with an Apstein net (0.17 m diameter opening, 55 μm mesh size except for two occasions with 10 μm mesh size; Table 1) on a weekly basis. The maximum sampling depth was 17 m to avoid contact of the Apstein net with the sediment traps. Sampling frequency was restricted to low numbers (usually 1–3) of net hauls per sampling day (usually every 8^th^ day) to avoid overfishing (Table 1). Zooplankton hauls were transported to shore directly after sampling where they were preserved with sodium tetraborate-buffered formalin (4% v/v) for counting and taxonomic analyses.

Depth profiles of salinity, temperature, pH, chlorophyll *a* (chl*a*), and photosynthetically active radiation (PAR) were measured with a CTD60M (Sea & Sun Technologies) on every sampling day. CTD casts within each mesocosm were typically conducted after sediment trap and water column sampling between 11 a.m and 3 p.m. (local time, Table 1). Sensor details of the CTD60M and data analysis procedures for salinity, temperature, and density were described by Schulz and Riebesell [27]. Correction of pH CTD data is described in the following section.

### 2.7 Sample processing, measurement, and analysis

Inorganic nutrient samples were filtered (cellulose acetate filters, pore size 0.45 μm; Whatman) directly after sampling and analyzed on the same day to avoid any possibility of concentration changes due to biological growth or decay. NO_3_^-^ + NO_2_^-^ (= NO_3_^-^/NO_2_^-^), Si(OH)_4_, and PO_4_^3-^ concentrations were measured with a SEAL Analytical QuAAtro AutoAnalyzer connected to JASCO Model FP-2020 Intelligent Fluorescence Detector and a SEAL Analytical XY2 autosampler. When NO_3_^-^/NO_2_^-^ and PO_4_^3-^ concentrations dropped below 0.1 μM during the phytoplankton bloom we switched to using the nanomolar system with a detection limit of 0.8 nM PO_4_^3-^ and 1.5 nM NO_3_^-^/NO_2_^-^, as reported by Patey et al. [28]. Both methods were used in parallel during the transition phase (days 31–41) for inter-comparison (average deltas of the measurements were ±4.5 nM for NO_3_^-^/NO_2_^-^ and ±2.9 nM for PO_4_^3-^). Both measurement approaches are based on spectrophotometric techniques developed by Murphy and Riley [29] and Hansen and Grasshoff [30]. Ammonium concentrations were determined fluorometrically following Holmes et al. [31]. Instrument precision was calculated from the average standard deviation (1σ) of triplicate samples (±0.02 μM for NO_3_^-^/NO_2_^-^, ±0.01 μM for PO_4_^3-^, ±0.05 μM for Si(OH)_4_ and ±0.01 μM for NH_4_^+^). Analyzer performance was monitored by recording baseline, calibration coefficients and slopes of the nutrient species over time. The variations observed throughout the experiment were within the analytical error of the methods.

DOC and total dissolved nitrogen (TDN) concentrations were measured by high-temperature catalytic oxidation on a Shimadzu TOC-VCPH analyzer with ASI-V auto sampler as described by Zark et al. [32]. The DON concentration was calculated as TDN–(NO_3_^-^/NO_2_^-^ + NH_4_^+^).

Carbonate chemistry samples were sterile-filtered (0.2 μm) with syringe filters into two separate 300 mL Schott DURAN glass bottles (pH, DIC) allowing an overflow of twice the bottle volumes. Sterile-filtered subsamples were stored at 4°C in the dark for a maximum of three days until analysis. DIC was determined by the colorimetric titration method established by Johnson et al. [33], with a precision of 3.0 μmol kg^-1^ (estimated from duplicates). The accuracy was set by calibration against certified reference materials, supplied by A. Dickson, Scripps Institution of Oceanography (USA). pH_T_ (total scale) was determined by a spectrophotometric method, based on the absorption ratio of the sulfonephthalein dye, *m*-cresol purple [34], with a precision of ~0.002 pH_T_ units and accuracy set by the equilibrium constants of the indicator. *p*CO_2_ and aragonite saturation state (Ω_aragonite_), were calculated from the combination of pH_T_ and DIC using CO2SYS (excel version 2.1 [35]) with the carbonate dissociation constants (K_1_ and K_2_) of Lueker et al. [36]. Input data included salinity, temperature, PO_4_^3-^ and Si(OH)_4_ data, where the latter two were from the previous sampling day in the few cases of missing nutrient data. For calculation of [HCO_3_^-^]/[H^+^] (proton concentration on free scale), measured pH_T_ was converted to the free scale using CO2SYS. pH profiles measured with the CTD were originally on the NBS scale. Thus, the mean pH_NBS_ averaged over the whole water column had an offset compared to spectrophotometrically measured pH_T_ values. We corrected this offset and recalibrated the CTD probe to the total pH scale by means of daily linear correlation between averaged water column pH_NBS_ measured in-situ and pH_T_ measured in the laboratory.

The PM samples were filtered using 200 mbar on cellulose acetate (biogenic silica (BSi)) or glass fiber filters (total particulate carbon (TPC), total particulate nitrogen (TPN)). All glass fiber filters and glass petri dishes for filter storage were precombusted (450°C, 6 h) prior to use, in order to remove residual organic matter. The PM samples were stored at -20°C in plastic (BSi) or glass Petri dishes (TPC, TPN) until analyses. Filters for BSi analysis were heated in 0.1 M NaOH (85°C, 135 minutes) to leach the particulate silica from the filters. After neutralizing with 0.05 M H_2_SO_4_, samples were analysed as dissolved silicate by spectrophotometry according to Hansen and Koroleff [37]. TPC and TPN samples were dried (60°C) over night and wrapped in tin foil before measurement with an elemental CN analyzer following Sharp [38].

Samples for pigment analysis were filtered on glass fibre filters (800 mL, gentle vacuum of ~200 mbar), carefully folded, and immediately frozen and stored at -80°C in cryovials. Pigments were extracted 4–7 months after sampling in actetone (90%) as described by Paul et al. [14]. Pigment extracts were used for analysis by means of reverse phase high performance liquid chromatography (HPLC, [39]) and their concentrations were calibrated with commercial standards. Contributions of individual phytoplankton groups to total chl*a* concentrations were calculated with CHEMTAX [40].

Flow cytometry samples for phytoplankton, bacteria, and virus abundances as well as microzooplankton samples (20–200 μm; mostly ciliates) were taken from the 10 L carboys directly after the boats returned from mesocosm sampling. Care was taken that the volume within the 10 L carboys was gently mixed before sub-sampling in order to avoid sinking bias. Bacteria and virus samples were immediately fixed with glutaraldehyde (0.5% v/v; 30 minutes), flash-frozen in liquid nitrogen, and stored at -80°C until analysis 4–7 months later with an Accuri C6 flowcytometer (BD Biosciences). For more details on the applied preservation and measurement procedures please refer to the protocols by Marie et al. [41] Larsen et al. [42], and Brussaard [43]. Phytoplankton samples were measured within three hours after sub-sampling with the Accuri C6 flowcytometer. Gates were set based on the forward scatter signal or red fluorescence signals except for the *Synechococcus* and cryptophyte–like groups where the orange instead of the red fluorescence signal was used to distinguish them from bulk phytoplankton. The size of different phytoplankton groups was determined by fractionation with a variety of polycarbonate filters (0.2, 0.8, 2, 3, 5, 8 μm) following Veldhuis and Kraay [44]. We distinguished between picoeukaryotes (Pico; 0.2–2 μm), small nanoautotrophs (Nano I; 0.2–8 μm), *Synechococcus*-like autotrophs (Synecho; 0.2–2 μm), and cryptophyte-like autotrophs (Crypto; 0.2–8 μm). Note that larger species like chain-forming diatoms which could potentially interfere with flow cytometry measurements were almost absent in the water column (as determined by light microscopy). It is therefore safe to assume that the flow cytometry measurements are representative for the size spectrum <200 μm. Autotrophs larger >200 μm, represented by the large diatom *Coscinodiscus* sp. were present in this experiment in considerable quantity. Their abundance was determined by photographing TPC/TPN and BSi, filters and counting cells manually using ImageJ. Microzooplankton samples were immediately fixed after sub-sampling with acidic Lugol solution and stored in 250 mL brown glass bottles until analysis. Based on chl*a* data it was decided to evaluate microzooplankton samples on a weekly basis until t73 and every second week thereafter (t73 –t103). Metazoan abundances (mostly copepods) from net haul samples (> 55 μm) were counted with a binocular microscope. Both microzooplankton and metazoan abundance were determined 3–12 months after sampling.

### 2.8 Data analyses

We applied ANalysis Of SIMilarity (ANOSIM; [45]) to determine whether significant differences in the plankton community composition were present between ambient (M1, M3, M5, M9, M10) and high CO_2_ mesocosms (M2, M4, M6, M7, M8). To account for the different scales in abundance of different organism groups, ranging from viruses to mesozooplankton, ANOSIM input data was “range normalized” as:

$$N_{normalized}=\frac{N}{N_{max}-N_{min}}$$ (1)

where *N* is the abundance of the individual groups, and *max* and *min* refer to the highest and lowest abundance measurement among the 10 mesocosms, respectively. The normalized data from 4 selected stages of succession was then used to generate four different Bray-Curtis dissimilarity matrices. The selected stages of succession were: (1) the beginning of the study (Table 3); (2) the individual peak chl*a* concentration for each mesocosm between t27 and t35 (S2 Table); (3) the individual peak chl*a* concentration for each mesocosm between t45 and t59 (S3 Table). Note that the days of highest chl*a* concentration (i.e. bloom peak) differed slightly among mesocosms so that plankton abundances used in the analysis were not always from the same days (see S2 and S3 Tables). (4) The post-bloom period for which we calculated the average values of plankton abundances for the period from t81 to t111 (S4 Table). A significant ANOSIM result (p < 0.05) indicates an effect of CO_2_ on the composition of the entire plankton community, i.e. that communities within the ambient CO_2_ mesocosms were more similar to each other than to the communities in the high CO_2_ mesocosms. Nonmetric multidimensional scaling (NMDS) was performed with the same Bray-Curtis dissimilarity matrices as the ANOSIM tests in order to visualize mesocosm clustering. Bray-Curtis dissimilarity matrices, NMDS results, ANOSIM results, and subsequent similarity percentage analysis (SIMPER, which point towards the components in the community that primarily drive the clustering) were assessed with the Fathom Matlab toolbox provided by Jones [46].

**Table 3:** Chemical and biological conditions at the beginning of the experiment.

		low CO_2_							high CO_2_								
parameter	average of	M1	M3	M5	M9	M10	mean low CO_2_	S.D. low CO_2_	M2	M4	M6	M7	M8	mean high CO_2_	S.D. high CO_2_	T-test	correlation with salinity (p-value)
salinity	t0, t1	29.03	29.16	29.26	29.26	28.91	29.12	0.15	29.16	28.96	29.11	29.16	29.19	29.12	0.09	0.93	
^a^DIC (μmol kg^-1^)	t-1	2072.2	2073.4	2079.3	2065.2	2073.4	2072.7	5.0	2066.0	2077.4	2075.7	2080.3	2075.9	2075.1	5.4	0.49	0.713
^a^pH_total_ (spectrophotometer)	t-1	8.096	8.059	8.046	8.038	8.063	8.061	0.022	8.058	8.063	8.062	8.046	8.037	8.053	0.011	0.51	**0.046**
NO_3_^-^ + NO_2_^-^ (μmol L^-1^)	t2—t11	6.89	6.70	6.81	6.73	6.80	6.79	0.07	6.73	6.68	6.75	6.76	6.69	6.72	0.04	0.12	0.605
Si(OH)_4_ (μmol L^-1^)	t2—t11	10.33	10.01	9.80	9.68	9.57	9.88	0.30	9.94	9.84	9.78	9.65	9.75	9.79	0.11	0.57	0.833
PO_4_^3-^ (μmol L^-1^)	t2—t11	0.76	0.76	0.76	0.76	0.77	0.76	0.007	0.74	0.74	0.76	0.76	0.75	0.75	0.010	0.11	0.915
DOC (μmol L^-1^)	t-1—t11	185	180	173	179	200	184	10	191	182	182	184	184	185	4	0.85	**0.033**
DON (μmol L^-1^)	t3—t11	16	16	16	15	15	15.6	0.4	15	16	16	16	16	15.7	0.4	0.96	0.873
POC (μmol L^-1^)	t-1—t3	13.8	14.2	16.1	14.7	13.3	14.4	1.1	13.2	14.3	14.4	15.7	15.5	14.6	1.0	0.77	0.056
PON (μmol L^-1^)	t-1—t3	2.0	2.1	2.3	1.8	1.9	2.0	0.2	2.0	2.0	2.0	2.3	2.1	2.1	0.2	0.56	0.331
chlorophyll *a* (ng L^-1^)	t-1	315	322	271	304	317	306	21	282	303	299	314	324	304	16	0.89	0.324
*Coscinodiscus (cells L^-1^)	t-1—t7	0.9	1.7	1.5	0.9	1.1	1.21	0.35	1.3	1.6	1.7	1.4	1.1	1.44	0.24	0.26	0.973
Prasinophytes (ng chl a L^-1^)	t-1	175	165	154	157	154	161	9	150	167	161	169	181	166	12	0.52	0.822
Dinophytes (ng chl a L^-1^)	t-1	26	19	17	16	16	19	4	16	19	22	14	18	18	3	0.75	0.410
Diatoms (ng chl a L^-1^)	t-1	35	43	21	52	63	43	16	30	35	29	48	51	39	10	0.65	0.467
Cryptophytes (ng chl a L^-1^)	t-1	75	92	76	74	82	80	8	83	78	84	79	70	79	5	0.81	0.640
Chrysophytes (ng chl a L^-1^)	t-1	4	4	3	5	3	4	1	3	3	4	4	4	3	0	0.50	0.273
*Pico (cells mL^-1^)	t-1	16980	18155	16980	18428	17750	17659	665	18942	19578	18393	19494	19472	19176	505	**0.00**	0.976
*Nano (cells mL^-1^)	t-1	1606	1333	1606	1520	1586	1530	116	1526	1779	1598	1882	1805	1718	150	0.06	0.692
*Crypto (cells mL^-1^)	t-1	178	160	178	170	152	167	12	190	181	166	271	193	200	41	0.15	0.502
*Synecho (cells mL^-1^)	t-1	887	916	887	951	834	895	43	828	934	834	891	1003	898	73	0.93	0.348
*Bacteria (cells mL^-1^)	t-1	817580	832681	869058	862507	895435	855452	30767	838855	881913	873826	870391	902652	873528	23080	0.32	0.693
*Virus like particles (ind mL^-1^)	t-1	10245000	8855714	10220714	9600714	12061429	10196714	1186756	9945000	8600714	9195000	10329286	10530714	9720143	807394	0.48	0.518
*Pseudocalanus* (male) (ind m^-3^)	t1	3752	2107	2742	4012	5772	3677	1401	2511	3203	3001	3579	4012	3261	570	0.56	0.152
*Pseudocalanus (female) (ind m^-3^)	t1	7071	3810	5339	6955	9380	6511	2086	5166	6898	5281	5743	6955	6009	865	0.63	0.072
*Pseud. Copepodites (ind m^-3^)	t1	3001	2559	2597	2039	3925	2824	704	2732	2771	2386	2771	2039	2540	323	0.43	**0.008**
*Nauplii (ind m^-3^)	t1	1655	2097	1924	1655	1732	1812	194	1597	2347	1789	1501	1655	1778	335	0.85	0.408
*Ciliates (cells mL^-1^)	t-1	2.9	3.1	2.5	3.5	1.9	2.8	0.6	2.5	2.9	3.1	2.0	2.2	2.5	0.5	0.46	0.477

Values are either the first measurements or an average of measurements from the first couple of days. For the community-based analysis we generally included functional groups rather than species. For copepods, we only included *Pseudocalanus* sp. here since this species strongly dominated the copepod community both in abundance and biomass (note, however, that “nauplii” includes copepod nauplii from all species since they were not distinguished taxonomically). Parameters marked with an asterisk were used in the ANOSIM/NMDS analysis (Fig 8A). Bold values indicate statistical significance (p = <0.05).

^a^ prior to the first CO_2_ addition

In addition to these multivariate tests, we performed Student’s *t*-tests for each measured variable separately. These were done with MS excel where we tested for equality of variance and normal distribution and then used an independent sample *t*-test (type 2 in case of homogeneity in variance, else type 3) to assess statistical significance (threshold p-value = 0.05).

## 3 Results and Discussion

### 3.1 Overview of important events, developments, and perturbations during the mesocosm study

#### 3.1.1 Restart of experiments due to technical difficulties

The conditions at our arrival in the fjord were challenging due to the presence of sea ice and its potential to damage the mesocosms. Prevailing easterly winds during the end of January and February led to stable air temperatures below 0°C (Fig 2A) and thus to continuing ice formation and drift. An upwelling event around the 10^th^ of February brought relatively warm (~5°C) and saline (~32) North Atlantic water to the surface melted the ice. We used this opportunity to close the mesocosms and start the experiment on February 12^th^, 2013 (t-25). However, the upwelling event was a curse and a blessing at the same time because the water entrapped in the mesocosm bags was effectively 300–600 kg heavier than the usual mixture of Baltic Sea and North Atlantic water (mean salinity ~25) which returned a few days after the upwelling. Even though we were aware of this problem and added extra buoyancy aids to the mesocosm floatation frames, we could not prevent the water from accumulating at the bottom of the bags. Consequently the mesocosm bags took on a pear-like shape and water was pressed out of the bags into the fjord through a weak point in the connection between the bags and the sediment traps. Thus, we had to stop the experiment on the 3^rd^ of March (t-6) and fix the sediment traps on shore because the mesocosm bags had lost a large fraction of the initially enclosed water (S1 Fig).

#### 3.1.2 Changes in the enclosed water mass

The second experiment started four days after the end of the first on 7^th^ March (t-2) and lasted for 113 days until the 28^th^ June (t111). The enclosed water mass had an average salinity of 29.12 (±0.11) which is close to the upper limit typically measured for the 0–19 m depth range in Gullmar Fjord [21]. Thus, we once more enclosed a water mass of primarily marine (North Sea) origin. This time, however, the relatively heavy water did not cause trouble as we were able to sustainably repair the leaking weak point of the sediment traps. Almost complete water exchange occurred between the opened mesocosm bags and the fjord during the period between the failed experiment and our second approach (t-6 –t-2). Only a rather small, yet unquantifiable, amount of water in the middle of the submersed mesocosm bags was not flushed out and was carried over into the successful second try. The influence of this carry over of water from the first experiment into the second one will be discussed in detail in section 3.2.1.

**Fig 3:**
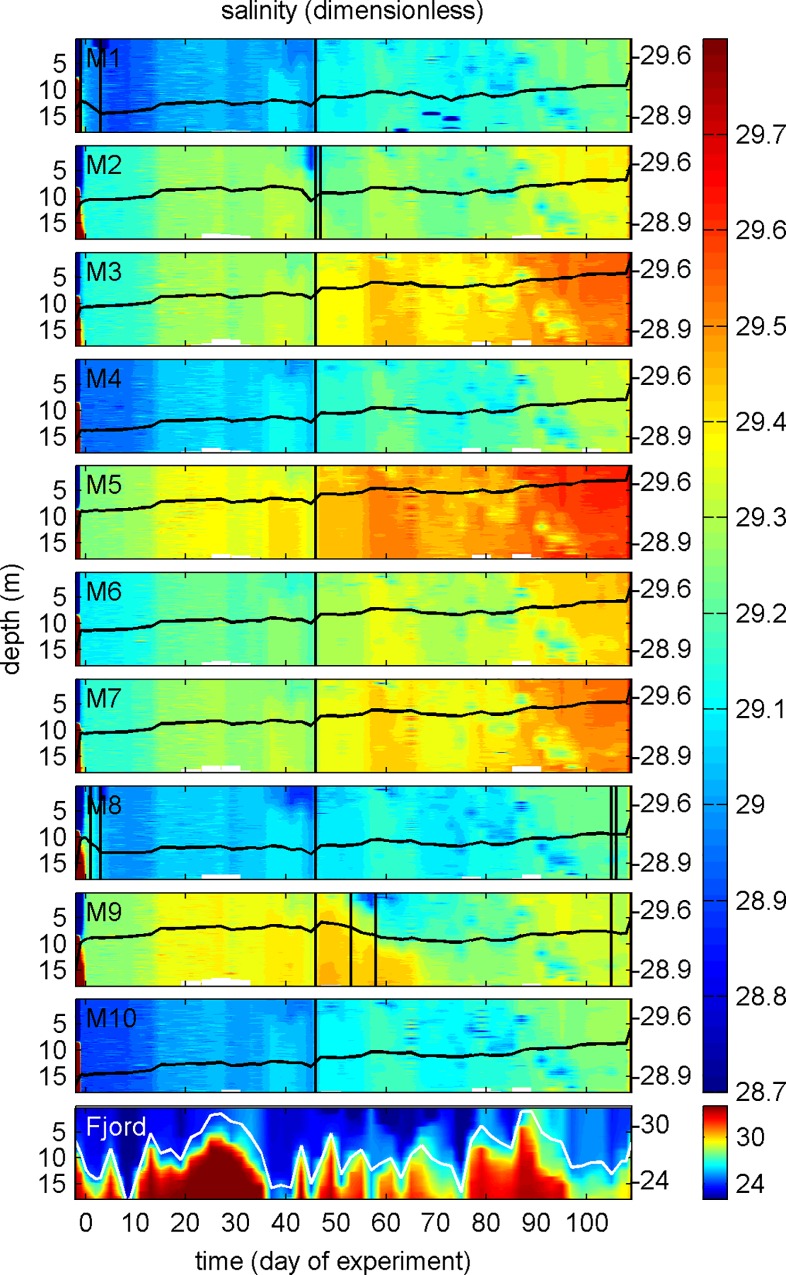
Salinity profiles over the course of the study. Note the different color coding for the fjord contour plot. Change of salinity averaged over the entire water column is represented by the black (or white in the case of the fjord) line plots on top of the contours with the corresponding additional y-axes on the right side. The vertical black lines on t46 mark the volume determination by brine (NaCl) addition. The other vertical black lines frame periods where we had small holes in the mesocosm bags (influx estimates given in Table 2).

Water exchange with the surrounding fjord stopped as soon as the sediment traps were attached and the upper part of the mesocosm bags pulled above the surface as explained in section 2.2. However, by cleaning the mesocosm bags with the cleaning ring we unfortunately created small cuts in the bags on 6 occasions during the experiment so that there was unintentional water exchange with the fjord until the cuts were repaired (Fig 3). Holes were sealed by divers with small rubber patches glued onto the outer side of the mesocosm bags. Detection of holes was difficult and sometimes took us several days of intense diving activity. In some instances they could not be detected from the outside so that it became necessary to dive into the mesocosm because it is much easier to spot the holes from the inside (inside diving events recorded in Table 1). Diving equipment was thoroughly cleaned before entering a mesocosm and we only used rebreathers in combination with dry suits and full face masks to minimize contamination and mixing of the enclosed water bodies.

**Fig 4:**
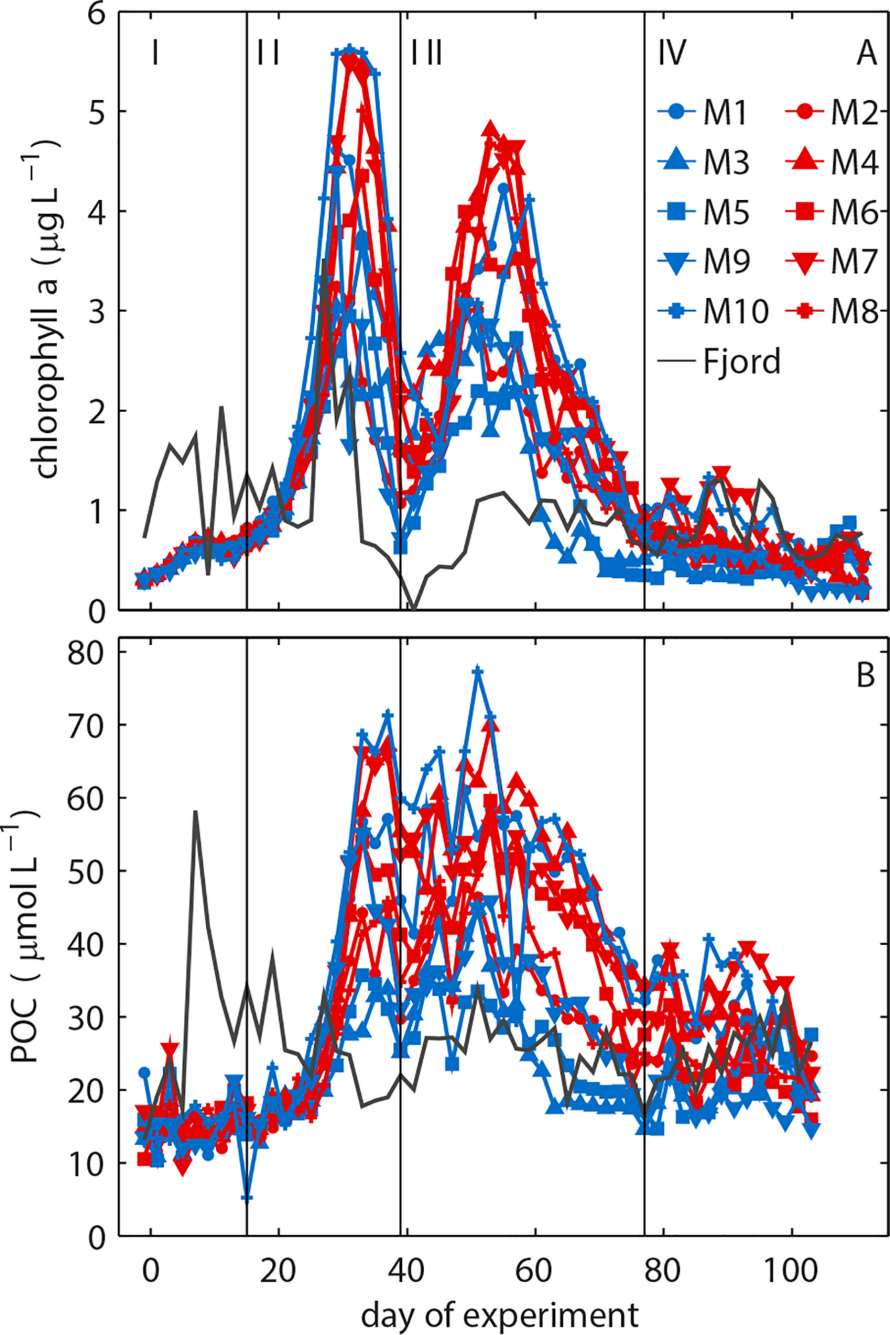
Development of (A) chl*a* and (B) POC concentrations over the course of the experiment. Roman numbers denote the different phases of the experiment.

Water exchange through holes was quantified by changes in mesocosm salinity. In the most extreme case (M8 between t0 and t5) it accounted for 3% of the total volume (all estimates given in Table 2). The impact of the water influx is difficult to assess but we did not observe anomalies in any of the measured parameters during or after these damages. Furthermore, mesocosms which were damaged did not have any fundamental differences in community succession (Fig 4). Hence, we tentatively conclude that the unintentional water influxes had a limited influence on the results presented here.

**Fig 5:**
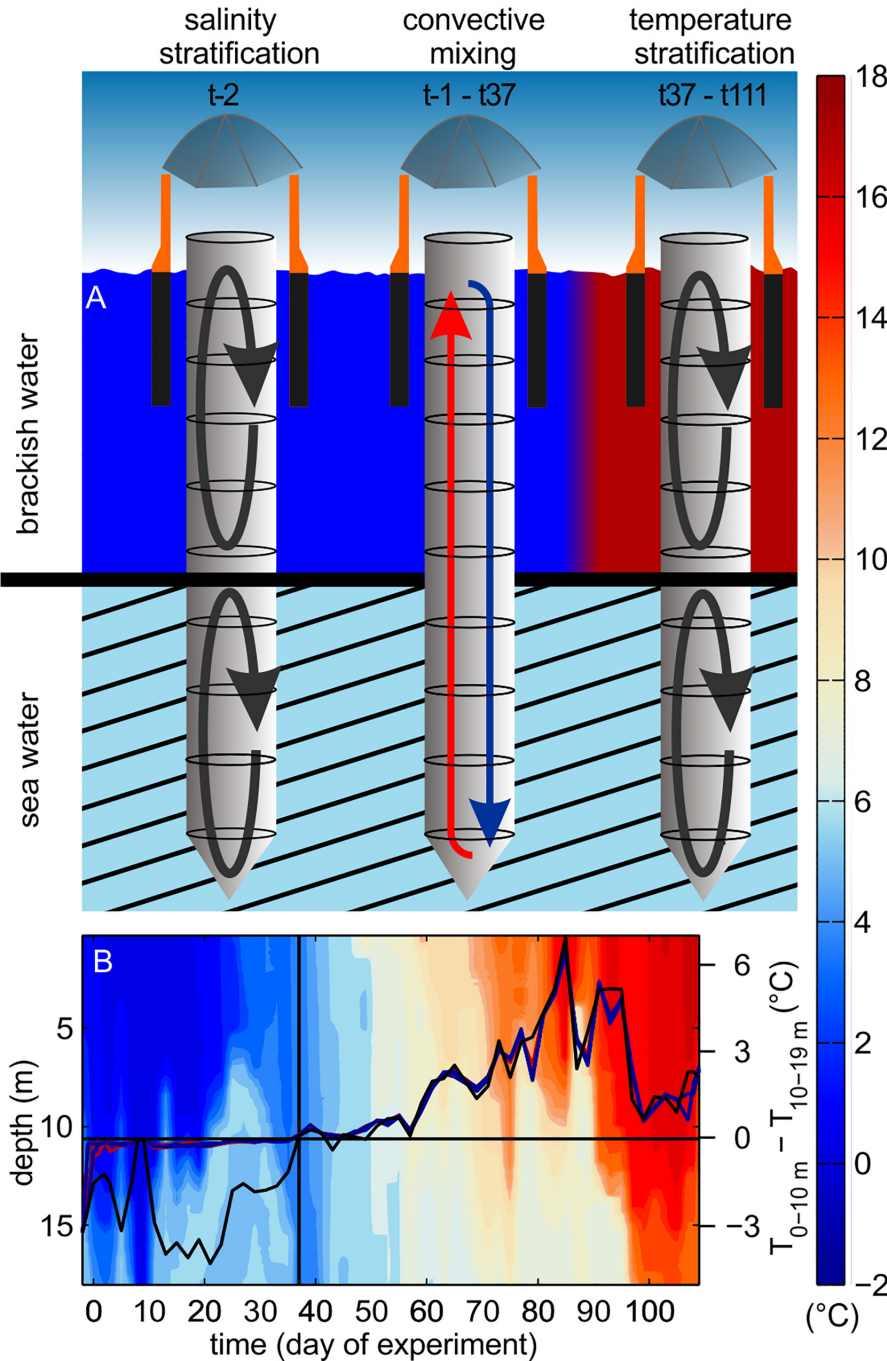
Changes in water column mixing in the course of the experiment. A salinity stratification prevented water column mixing at the beginning of the study (t-2). Convective mixing was initiated after homogenizing water column salinity. Convection was sustained until t37 by saline North Sea water which was warmer than the fresher surface water. (B) Surface water temperature rose above that of the deep water after t37 thereby establishing temperature stratification and terminating convective mixing.

**Fig 6:**
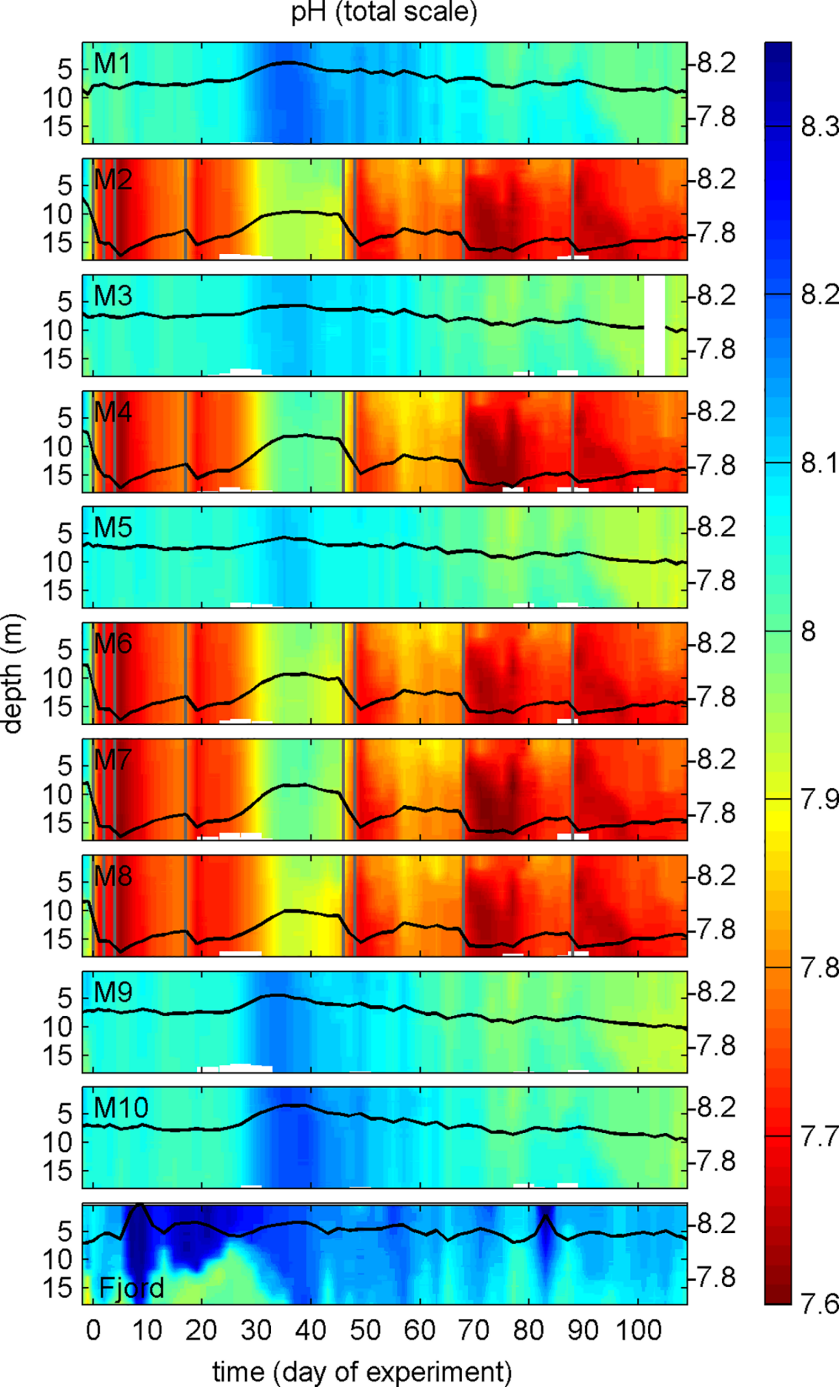
pH_T_ depth profiles at *in-situ* temperature over the course of the study. Change of pH_T_ averaged over the entire water column is represented by the black line plot on top of the contours with the corresponding y-axes on the right side. The vertical grey lines signify days of carbonate chemistry manipulation by additions of CO_2_-aerated water.

The water column was mixed immediately after the second closing procedure on t-2 in order to break down the existing halocline. The salinity homogenization initiated a strong convective mixing inside the bags, as the deeper water in the fjord (North Atlantic water) was warmer than the fresher top layer thereby constantly heating the lower parts of the mesocosms (Fig 5). Since the salinity stratification was absent inside the mesocosms after mixing, the water at the bottom of the mesocosm, warmed by the adjacent fjord, could rise to the surface where it was cooled by the cold, low salinity fjord water (Fig 5). This convection cell thoroughly homogenized the water column (Figs 3 and 6) until mid-April (t37), the time when surface water temperatures exceeded those of the bottom water, and established a thermocline which prevailed until the end of the experiment (Fig 5).

The masses of the water enclosed within the ten mesocosms were determined on t46. On this particular date, masses ranged from 55.9 tons in M2 to 47.5 tons in M3 (Table 2). Note, however, that mass changed slightly over the course of the experiment due to evaporation, rain, sampling, seeding and the unintentional water exchange through holes.

#### 3.1.3 Different phases of bloom development

**Fig 7:**
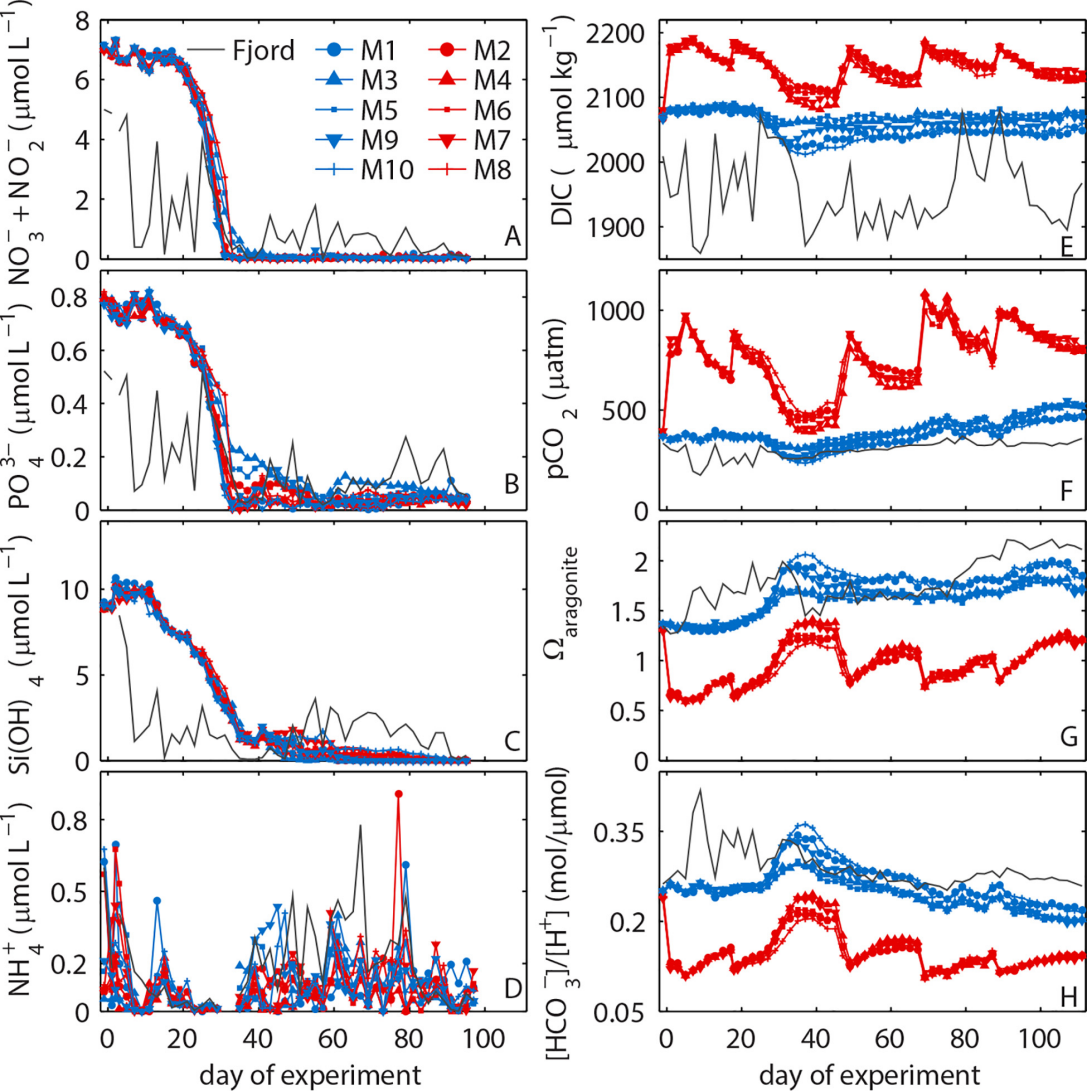
Water column integrated (0–17 m) inorganic nutrient concentrations and carbonate chemistry conditions inside the mesocosms and in the surrounding fjord water.

Surface irradiance at the beginning of the study was relatively low (Fig 2B) and convective water column mixing homogenized phytoplankton distribution over the entire water column. The lack of stratification did, however, not inhibit the growth of phytoplankton. Chl*a* concentrations increased steadily from the first day until t10 where a short depression was observed before growth regained momentum and led to the first chl*a* peak between t29 to t35 with mesocosm-specific intensity (highest in M10 and lowest in M3; Fig 4A). Scanning electron microscopy samples revealed that the most important species contributing to the chl*a* build-up were the small (2–5 μm) silicifying species *Arcocellulus* sp., *Minidiscus* sp. (both diatoms), and *Tetraparma* sp. (Chrysophyte) as well as the very large (>200 μm) diatom *Coscinodiscus* sp. The first bloom was fueled by inorganic nutrients upwelled during winter and enclosed in the mesocosms at the beginning of the study. Initial concentrations of NO_3_^-^/NO_2_^-^, PO_4_^3-^, and Si(OH)_4_ were ~6.8, ~0.7 and 9.85 μmol L^-1^, respectively (Table 3; Fig 7), which is within the range typically observed in this region before the spring bloom [47]. The collapse of the phytoplankton spring bloom was not initiated by the abrupt end of convective mixing on t37 as chl*a* decrease began 2 to 7 days earlier. Instead, it is most likely attributable to aggregation and subsequent sedimentation as well as ongoing grazing pressure at the point where NO_3_^-^/NO_2_^-^ concentrations ran into limitation (Fig 7A). PO_4_^3-^ was also low at peak bloom but concentrations remained far above the detection limit (0.8 nmol L^-1^) and fluctuated at a low level (max 0.2 μmol L^-1^) from around t35 onwards (Fig 7B). The Si(OH)_4_ decline was more linear than that of NO_3_^-^/NO_2_^-^ and PO_4_^3-^ (Fig 7C). Detection limit was reached quite shortly after peak bloom in some mesocosms (e.g. M3, M5), while it took up to forty days longer in others (e.g. M10).

A second phytoplankton bloom developed directly after the first collapsed. Bloom magnitude was on average slightly lower than in the first bloom with peak1/peak2 chl*a* ratios ranging from 1.5 (M10) to 0.9 (M3). The most important taxa contributing to the second bloom were small (2–5 μm) diatoms (*Minidiscus* sp., *Arcocellulus* sp.), a large variety of small green flagellates (0.8–5 μm) and *Coscinodiscus* sp. Chl*a* build-up was fueled by remineralized nutrients as new nutrients upwelled by winter mixing and available at the beginning of the experiment were depleted at this time point. DON and NH_4_^+^concentrations did not noticeably change during the second bloom suggesting that nutrients required for bloom development were remineralized and directly transferred into phytoplankton biomass without transient accumulation in the dissolved organic and inorganic nutrient pools (Fig 7D; [32]). Stable post-bloom conditions were established in the aftermath of the second bloom. They were characterized by low chl*a* concentrations and an intensifying temperature stratification of the water column (Figs 4A and 5B).

POC concentrations increased with chl*a* during the first bloom but did not follow its decline thereafter (Fig 4B). Instead, POC remained at elevated levels and bridged the chl*a* gap between the two blooms (compare Fig 4A and 4B). This suggests that most POC generated by phytoplankton was retained in the water column in the form of senescent phytoplankton detritus and/or routed in heterotrophic biomass. The chl*a* decline after the second bloom and the low level stagnation during the post-bloom period was reflected in POC concentrations (Fig 4B). Similar to chl*a*, there was also a noticeable variance in POC trends between mesocosms. In the first bloom between t30 and t40, for example, POC increased to up to 71 μmol L^-1^ in M10 but only reached a maximum of 34 μmol L^-1^ in M3. Reasons for such large differences in chl*a* and POC development between mesocosms are unclear at present but there is some evidence that they originate from differences in the plankton community enclosed at the beginning of the study (see section 3.2.1 for further details).

Based on the development in chl*a* concentrations described above we define four major phases of the winter-to-summer succession in the mesocosms (Fig 4). Phase I lasted from the beginning of the experiment until t16 and encompasses the period before the major chl*a* build-up of the spring bloom. Phase II lasted from t17 until t40 and includes the major chl*a* build-up and decline of the spring bloom. Phase III started on t41 and ends after the second chl*a* peak has gone on t77. Phase IV covers the post-bloom phase and ends with the experiment on t111.

#### 3.1.4 Carbonate chemistry conditions

Injections of CO_2_ enriched seawater into the designated high CO_2_ mesocosms (M2, M4, M6, M7, M8) on t-1 and t0 elevated the DIC concentration from 2075 (±5) to 2186 (±3) μmol kg^-1^ (Fig 7E). This change increased *p*CO_2_ from 385 (± 9) to 960 (±10) μatm (Fig 7F) and reduced pH_T_ from 8.045 (±0.009) to 7.674 (±0.004) (Fig 6). Ω_aragonite_ and the substrate-inhibitor ratio for calcification ([HCO_3_^-^]/[H^+^]) are two specifically relevant carbonate chemistry parameters for calcifying organisms as they control post-production dissolution of aragonite (Ω_aragonite_) and characterize the ability of the carbonate system to support high CaCO_3_ formation rates ([HCO_3_^-^]/[H^+^]; [48]. Ω_aragonite_ dropped below 1 (~0.6) upon CO_2_ addition so that seawater in the low pH treatments was corrosive for aragonite (Fig 7G). [HCO_3_^-^]/[H^+^] declined from ~0.25 to ~0.12 mol/μmol (Fig 7H) suggesting that biotic formation of CaCO_3_ was more challenging under high CO_2_.

The mesocosms were an open system and gas exchange occurred at the air-sea boundary layer [49]. Thus, DIC concentrations needed to be readjusted in the high CO_2_ treatment on 5 occasions during the experiment in order to compensate for CO_2_ loss to the atmosphere (Fig 6; Table 1). The differences in carbonate chemistry conditions between the high and ambient CO_2_ treatment were variable in the course of the experiment but at no point overlapped (Fig 7). DIC concentrations declined in both treatments during the spring bloom in phase II primarily due to DIC uptake by photoautotrophs. In the aftermath of the bloom, DIC remained relatively stable with only some gas-exchange driven fluctuations in the high CO_2_ treatment. The absence of a DIC decrease in the ambient CO_2_ treatment during the second bloom suggests that inorganic carbon used for autotroph growth in phase III probably originated from respired biomass and thus was supplied by heterotrophs. The *p*CO_2_ trends reflect the changes in DIC with the exception of a continuous increase in the second half of the study caused by the warming of water inside the mesocosms. Ω_aragonite_ and [HCO_3_^-^]/[H^+^] increase until the peak of the spring bloom. Ω_aragonite_ remained relatively stable thereafter because the influence of increasing DIC is counterbalanced by increasing temperature. Corrosive conditions for aragonite were present during most of the time in the high CO_2_ treatment (Fig 7G). In contrast to Ω_aragonite_, [HCO_3_^-^]/[H^+^] is insensitive to changing temperature but becomes smaller with decreasing pH [48]. It therefore constantly decreases after the spring bloom (Fig 7H) suggesting that carbonate chemistry conditions for calcification deteriorated from spring to summer.

Vertically, carbonate chemistry conditions were homogenous until the end of convective mixing on t37 (Fig 6). Mildly stratified conditions developed thereafter with generally higher pH_T_ (lower *p*CO_2_) in the upper mixed layer of the high CO_2_ treatment and generally lower pH_T_ (higher *p*CO_2_) in the upper mixed layer of the ambient CO_2_ treatment (Fig 7). Differences in vertical pH zonation between the two treatments were due to the opposing direction of air-to-sea gas exchange; net CO_2_ outgassing was dominant in the high CO_2_ mesocosms while net in-gassing was persistent under ambient CO_2_ conditions except for phase IV.

### 3.2 Plankton community structure

#### 3.2.1 Influence of initial differences in the plankton community upon their succession

**Fig 8:**
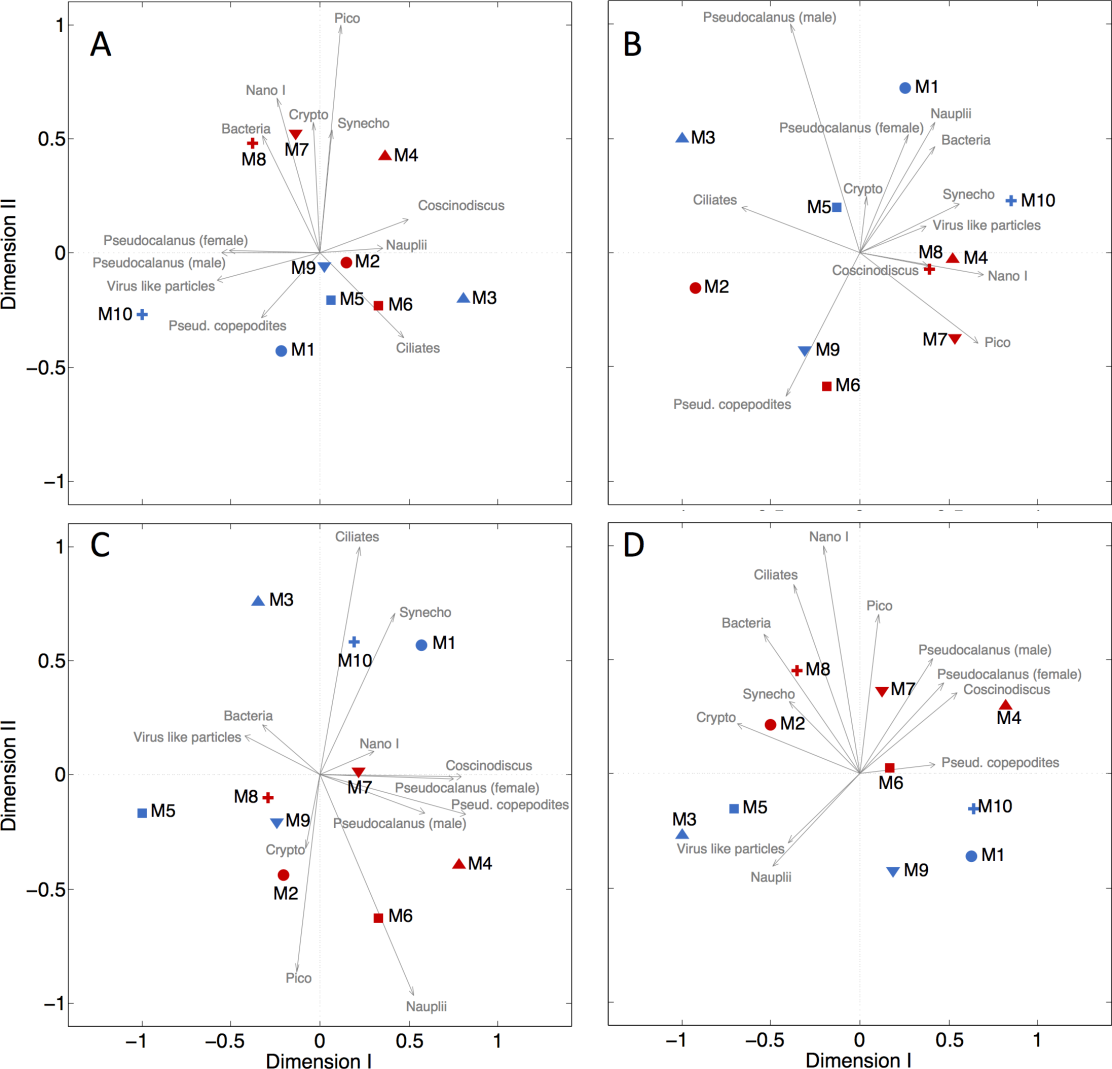
NMDS analysis of plankton community composition based on Bray-Curtis dissimilarities from (A) the beginning of the experiment (Stress = 0.0452), (B) peak chl*a* concentrations during the first bloom (Stress = 0.0269), (C) peak chl*a* concentrations during the second bloom (Stress = 0.0831), and (D) during the post-bloom period (Stress = 0.0138). Significant clustering (ANOSIM p = 0.039) between ambient and high CO_2_ mesocosms was only observed during the second bloom (C). The underlying data implemented in the analysis are shown in Table 3 and S2, S3 and S4 Tables.

The composition of the plankton community enclosed at the beginning of an experiment influences its subsequent succession. A detailed summary of the initial conditions revealed broadly similar conditions in most biogeochemical and community-related parameters at the level of detail investigated here (Table 3). Heterogeneity among the ten mesocosms was primarily found in those parameters with lower measurement precision or where measurements were close to detection limit (e.g. copepod and diatom abundance; Table 3). Correlations between biogeochemical or community-based parameters and salinity were used to assess whether differences at the beginning of the study could be due to enclosure of different water masses. These correlations were only in 3 out of 27 cases significant (Table 3) indicating that there seems to be no systematic difference among mesocosms related to differential water exchange before closing. Variability among mesocosms was generally larger in the ambient CO_2_ treatment than in the high CO_2_ treatment, with the standard deviation (SD) being higher in 22 out of 28 measured parameters (Table 3). This is also reflected in NMDS analysis where the spread among ambient CO_2_ mesocosms looks higher than in the high CO_2_ treatment (Fig 8A).

Results from the ANOSIM test suggested that there was no significant difference between the plankton communities in the high CO_2_ and low CO_2_ mesocosms at the beginning of the study (R = 0.13; p = 0.2). To support the ANOSIM results we performed *t*-tests with each of the measured parameters separately. The *t*-tests generally support the ANOSIM result (no significant difference among treatments in 27 out of 28 parameters) with the exception of picoeukaryotes where the *t*-test implied significantly (*p* = 0.0018) higher abundance in the high CO_2_ treatment (Table 3). The initial difference in picoeukaryote abundance uncovered by the *t*-test can be explained by the outcome of the first (failed) experiment which was terminated after 19 days due to technical problems (section 3.1.1). In this preceding experiment, we observed a strong positive effect of CO_2_ on the abundance of picoeukaryotes (S2A Fig). This outcome was partially transferred into the second experiment because of the incomplete water exchange within the mesocosms during the four days between the two studies (S2B Fig, see also section 3.1.1). Based on this evidence we refrain from using the ANOSIM result (which is based on multivariate analysis of all community parameters) and follow the univariate *t*-test analysis which suggested that both mesocosm treatments showed no significant difference at the beginning of the study except for the picoeukaryote abundance.

Some of the patterns recorded at the beginning of the study seemed to diminish over the course of the experiment whereas others were conserved. For instance, the higher variability of community structure in the ambient CO_2_ treatment (higher SD in 22 out of 28 measured parameters, see above) was still present during the first bloom (SD higher in 9 out of 12 parameters, S2 Table, Fig 8B) but this pattern vanished in the second bloom (SD higher in 6 out of 12 parameters, S3 Table, Fig 8C) and in the post-bloom period (SD higher in 5 out of 12 parameters, S4 Table, Fig 8D). The initially large difference between M3 and M10 (both ambient CO_2_ replicates; Fig 8) seemed to be conserved, a feature which was also observed during the peak of the bloom in phase II and then again in the post-bloom period, after having a short interval of relatively similar conditions in phase III. These two mesocosms also had a particularly different development in chl*a* concentrations (Fig 4A), POC concentrations (Fig 4B), and many other measured parameters (shown in the more specialized publications of this PLOS collection; S1 Table) which strengthens the impression that differences in the plankton community enclosed at the beginning of the study may in part explain the variability observed during its subsequent succession.

A critical aspect in this context is the above-mentioned significant higher abundance of picoeukaryotes in the high CO_2_ treatment. Due to this remnant from the first experiment we cannot fully clarify to what extent the picoeukaryote response in the second experiment was preset by the initial conditions and to what extent the response developed over the course of the experiment. Several lines of evidence, however, suggest that initial difference was of minor importance for the responses later in the succession. Initial differences were on average ~1500 cells mL^-1^ which is less than 9% of the total population (Table 3). Thus, only 9% of the population in the ambient CO_2_ mesocosms would have needed to divide in order to equalize the initial difference. This could be achieved within hours since picoeukaryotes are able to divide more than twice per day [50]. Therefore, it is not surprising that this significant difference between ambient and high CO2 treatment is lost already within phase I (indicated by a switch of a t-test *p*-value from 0.04 on t15 to 0.23 on t17). A loss of the initial difference in picoeukaryotes during phase I strongly suggests that the re-establishment of a positive CO_2_ effect during phase III (S3 Table) was formed by processes taking place during the succession and not as a result of initial differences. However, even in the unlikely case that the phase III observations were caused by the initial differences, they would still be CO_2_-induced (S2 Fig). Thus, the carry-over of the positive CO_2_ effect on picoeukaryote abundance would not negate the conclusions made in this study.

#### 3.2.2 Restructuring of the plankton community by ocean acidification

A major motivation of this study was to test whether simulated end of the century CO_2_ concentrations can restructure entire plankton communities on a natural winter-to-summer succession. Succession patterns can be investigated at different degrees of functional or taxonomic resolution. The present analysis included various plankton types with a relatively broad functional spectrum but did not account for CO_2_ effects within functional groups. CO_2_ effects on or within specific functional groups (e.g. picoautotrophs) or taxonomic entities (e.g. crustaceans) will be investigated in more targeted studies presented within the framework of this special issue (S1 Table). At the level of detail investigated in the present work, a significant CO_2_ effect on plankton community structure was subtle and only detectable during the second phytoplankton bloom (phase III). Here, the main drivers of the CO_2_-induced community restructuring were nauplii, bacteria, and picoeukaryote abundances (Fig 8C; S3 Table). No CO_2_ effects were detected in any of the three other stages of succession (phase I, II, IV; Fig 8) which leads to two key questions: Why was the CO_2_ effect restricted to phase III? And, how was this CO_2_ effect on community composition generated?

To answer this we need to characterize the stage of succession at which the CO_2_ effect occurred. The second phytoplankton bloom was profoundly different to the first one as it was fueled by remineralized nutrients (section 3.1.2). Thus, essential resources for autotrophic growth needed to be provided by, or extracted from, various sources within the food-web. In contrast, no upstream ecosystem processes were necessary during the first bloom where upwelled inorganic nutrients were naturally available. A putatively more complex ecosystem structure during the second bloom may consequently have provided more “contact points” for altered carbonate chemistry to induce community restructuring. Conversely, a comparatively low ecosystem complexity during the first bloom where a few dominant phytoplankton species outgrew the others may have led to a fairly one-dimensional nutrient flux through the food-web. In such a bloom setting, altered CO_2_ conditions may be less likely to significantly affect bloom development because only a few (often only one) fast growing species are involved.

The obvious problem with this line of reasoning is the inconsistency of results between phase III and phase IV (the post-bloom phase) where the plankton community was similarly fueled by regenerated nutrients. However, the absence of a detectable CO_2_ effect during phase IV (Fig 8D) is probably not surprising due to the following. In phase III we analyzed a defined event (peak of the second bloom) whereas we averaged each parameter over the entire post-bloom period in phase IV (S4 Table). Averaging over phase IV was necessary because there was no clear event suitable for a more focused analysis. Hence, we may have missed a potential CO_2_-induced community restructuring due to the averaging of a large time period. Furthermore, we also noticed that fluctuations of taxonomic group or species abundances over time seemed to increasingly desynchronize among replicates the longer the experiment lasted. Accordingly, later in the experiment it became more and more difficult to uncover CO_2_ effects because even if they were present they may have occurred at different days in the five replicates. Uncovering such temporal mismatches seems to be one of the major challenges in long-term mesocosm studies but being able to resolve this problem is essential in order to avoid an inflation of type II errors (concluding there is no CO_2_ effect on the plankton community even though there is one).

## 4 Conclusion and Outlook

In this experiment we investigated the influence of realistic end-of-the-century carbonate chemistry conditions on a natural winter-to-summer plankton succession in a coastal pelagic ecosystem for a period of 113 days. An examination of key biogeochemical variables and the plankton community composition before CO_2_ treatment revealed broadly similar starting conditions among replicates. However, some of the variability present at the beginning seemed to be conserved in the succession pattern, which suggests that consideration of starting conditions is necessary to understand the temporal dynamics of plankton community composition. Furthermore, we noticed that initial differences in combination with the variability introduced in the course of the succession makes it increasingly difficult to detect CO_2_-induced effects later in the experiment which is a considerable complication of these kinds of long-term studies.

At the level of detail investigated in this study we found that CO_2_-induced changes in plankton community composition were generally subtle and detected only in a succession stage where a phytoplankton bloom was fueled by remineralized nutrients. This finding agrees with two other recent studies in different oceanographic regimes which also reported the most noticeable CO_2_ effects to occur at limiting inorganic nutrient concentrations [14,51]. Since most published OA experiments with plankton communities were conducted in relatively eutrophic settings, we may thus far have missed many potential CO_2_ effects. We should therefore focus on settings where plankton communities are fueled by regenerated nutrients in future OA studies.

## Supporting Information

S1 Fig Underwater photograph (~8 m depth) of a mesocosm at the end of the first (failed) experiment where we enclosed seawater with a considerably higher salinity than usually experienced in the fjord. The heavy water was accumulating at the bottom of the mesocosm and leaked out of the bags into the fjord through a weak point in the connection between the bag and the sediment trap. Seen here is the upper part of the bag that became compressed as a consequence of water leakage at the bottom. Sampling was impossible at that point because the sampling gear no longer fitted into the mesocosm bag.
(DOCX)

S2 Fig (A) Development of picoeukaryote abundance in the first experiment (12^th^ February until the 3^rd^ March). The two grey lines frame the period of CO_2_ addition. High CO_2_ mescosms (warm colors: M2, M4, M6, M7, M8) reached an average *p*CO_2_ of 1063 (±15) μatm on t-16. Ambient CO_2_ mesocosms (cold colors: M1, M3, M5, M9, M10) were left unperturbed with an average *p*CO_2_ of 371 (±1.5) μatm. A pronounced positive effect of elevated *p*CO_2_ emerged after t-13. (B) pH_NBS_ CTD profiles from the very beginning of the second study (7^th^ of March, t-2), directly after closing the mesocosms but before mixing them with compressed air. pH_NBS_ profiles reveal that some of the high CO_2_ water from the first experiment was still present in some mesocosms (mainly M7 and M8) at the beginning of the second approach, even though mesocosm bags were completely under water and the sediment traps were removed during the 4 days in between the two studies.
(DOCX)

S3 Fig Temperature profiles over the course of the study. Changes in temperature averaged over the entire water column are represented by the white line plots on top of the contours with the corresponding y-axes on the right side. The black lines at t37 mark the end of convective mixing (See also Fig 5).
(DOCX)

S1 Table Contributions intended to be published within the framework of the BIOACID II long-term mesocosm study. Note that two studies [16,32] have already been published before initiating the PLOS collection.
(DOCX)

S2 Table Abundance of individual plankton groups during peak chl*a* concentrations in phase II. For the community-based analysis we generally included functional groups rather than species. For copepods, we only included *Pseudocalanus* sp. here since this species strongly dominated the copepod community both, numerically and in terms of biomass. (Note, however, that “nauplii” includes copepod nauplii from all species since they were not distinguished taxonomically). This data was used for ANOSIM/NMDS analysis (Fig 8B).
(DOCX)

S3 Table Abundance of individual plankton groups during peak chl*a* concentrations in phase III. For the community-based analysis we generally included functional groups rather than species. For copepods, we only included *Pseudocalanus* sp. here since this species strongly dominated the copepod community both, numerically and in terms of biomass. (Note, however, that “nauplii” includes copepod nauplii from all species since they were not distinguished taxonomically). This data was used for ANOSIM/NMDS analysis (Fig 8C).
(DOCX)

S4 Table Abundance of individual plankton groups during the post bloom period in phase IV. For the community-based analysis we generally included functional groups rather than species. For copepods, we only included *Pseudocalanus* sp. here since this species strongly dominated the copepod community both, numerically and in terms of biomass. (Note, however, that “nauplii” includes copepod nauplii from all species since they were not distinguished taxonomically). This data was used for ANOSIM/NMDS analysis (Fig 8D).
(DOCX)
