# Supplementary material for: Influence of Ocean Acidification on a Natural Winter-to-Summer Plankton Succession: First Insights from a Long-Term Mesocosm Study Draw Attention to Periods of Low Nutrient Concentrations
Source: PLoS One. 2016 Aug 15;11(8):e0159068. doi: 10.1371/journal.pone.0159068 (PMC4985126; doi:10.1371/journal.pone.0159068)
Supplement: S1 Fig — The heavy water was accumulating at the bottom of the mesocosm and leaked out of the bags into the fjord through a weak point in the connection between the bag and the sediment trap. Seen here is the upper part of the bag that became compressed as a consequence of water leakage at the bottom. Sampling was impossible at that point because the sampling gear no longer fitted into the mesocosm bag. (DOCX) [file pone.0159068.s001.docx]

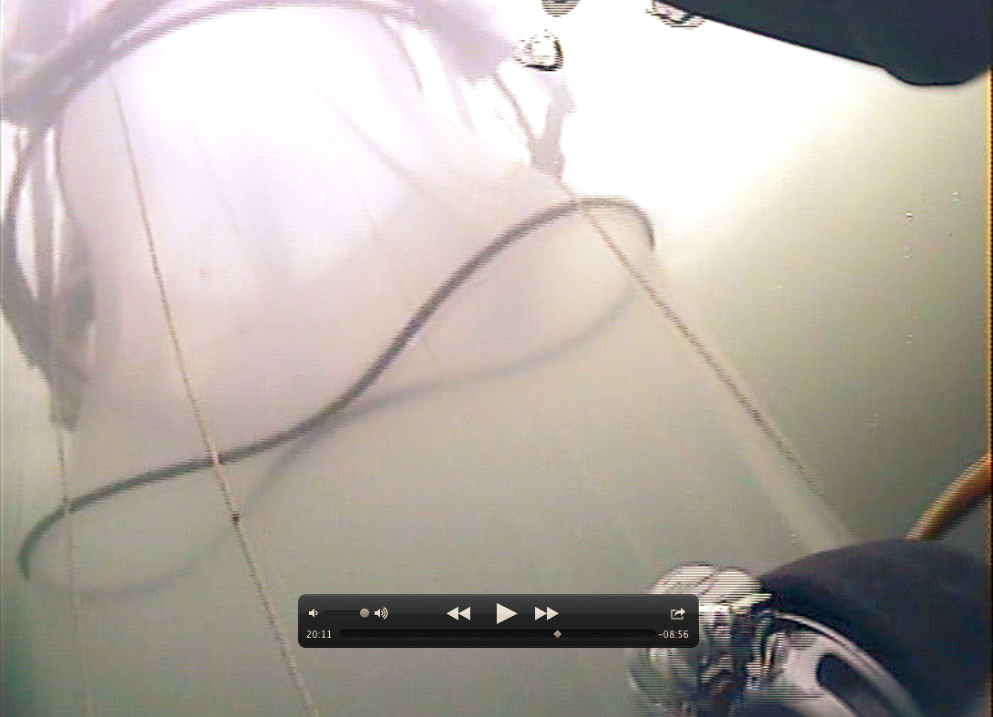


**S1 Fig.** Underwater photograph (~8 m depth) of a mesocosm at the end of the first (failed) experiment where we enclosed seawater with a considerably higher salinity than usually experienced in the fjord. The heavy water was accumulating at the bottom of the mesocosm and leaked out of the bags into the fjord through a weak point in the connection between the bag and the sediment trap. Seen here is the upper part of the bag that became compressed as a consequence of water leakage at the bottom. Sampling was impossible at that point because the sampling gear no longer fitted into the mesocosm bag.
