# Supplementary material for: Influence of Ocean Acidification on a Natural Winter-to-Summer Plankton Succession: First Insights from a Long-Term Mesocosm Study Draw Attention to Periods of Low Nutrient Concentrations
Source: PLoS One. 2016 Aug 15;11(8):e0159068. doi: 10.1371/journal.pone.0159068 (PMC4985126; doi:10.1371/journal.pone.0159068)
Supplement: S2 Fig — (A) Development of picoeukaryote abundance in the first experiment (12th February until the 3rd March). The two grey lines frame the period of CO2 addition. High CO2 mescosms (warm colors: M2, M4, M6, M7, M8) reached an average pCO2 of 1063 (±15) μatm on t-16. Ambient CO2 mesocosms (cold colors: M1, M3, M5, M9, M10) were left unperturbed with an average pCO2 of 371 (±1.5) μatm. A pronounced positive effect of elevated pCO2 emerged after t-13. (B) pHNBS CTD profiles from the very beginning of the second study (7th of March, t-2), directly after closing the mesocosms but before mixing them with compressed air. pHNBS profiles reveal that some of the high CO2 water from the first experiment was still present in some mesocosms (mainly M7 and M8) at the beginning of the second approach, even though mesocosm bags were completely under water and the sediment traps were removed during the 4 days in between the two studies. (DOCX) [file pone.0159068.s002.docx]

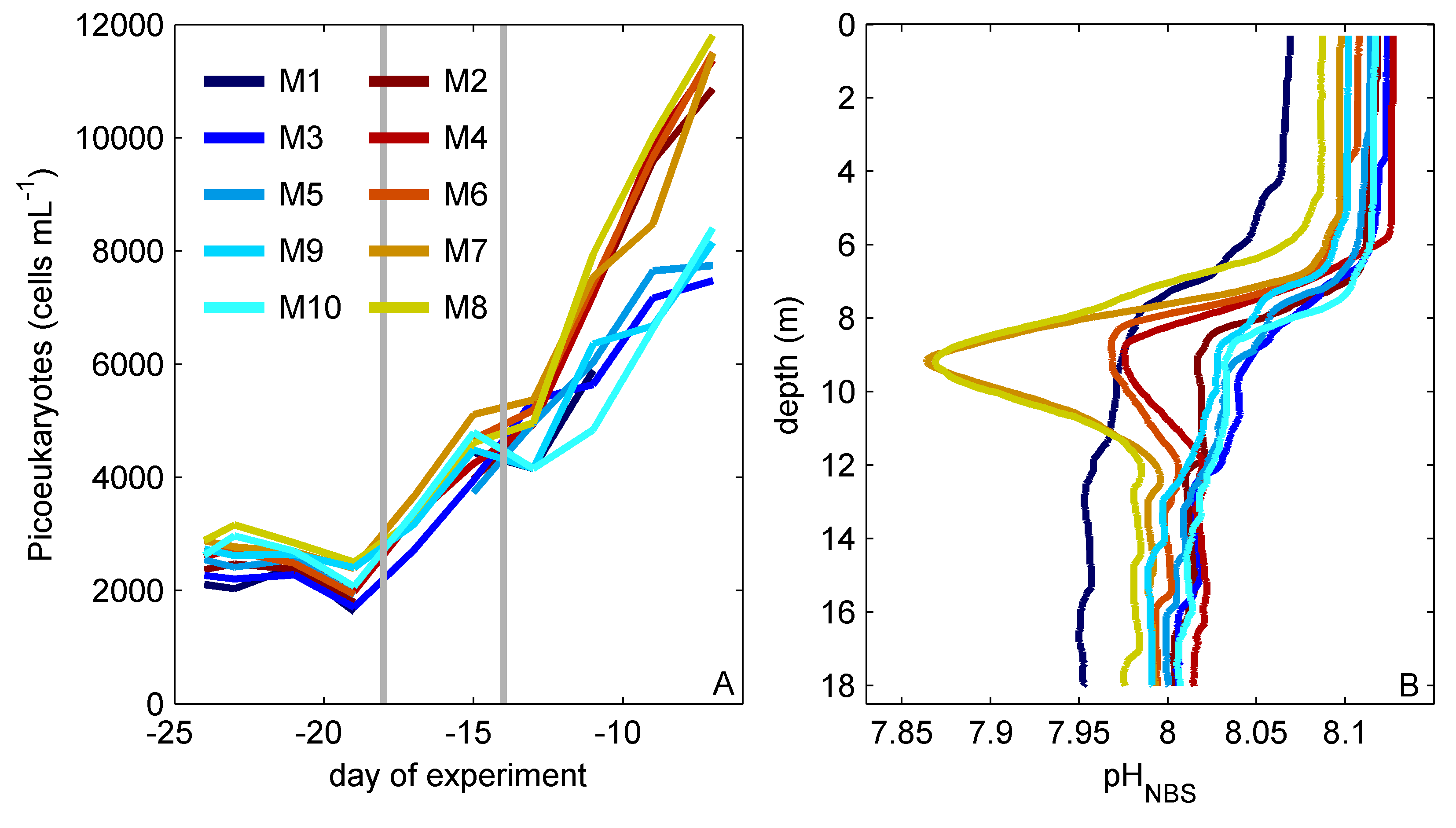


**S2 Fig.** (A) Development of picoeukaryote abundance in the first experiment (12^th^ February until the 3^rd^ March). The two grey lines frame the period of CO_2_ addition. High CO_2_ mescosms (warm colors: M2, M4, M6, M7, M8) reached an average *p*CO_2_ of 1063 (±15) µatm on t-16. Ambient CO_2_ mesocosms (cold colors: M1, M3, M5, M9, M10) were left unperturbed with an average *p*CO_2_ of 371 (±1.5) µatm. A pronounced positive effect of elevated *p*CO_2_ emerged after t-13. (B) pH_NBS_ CTD profiles from the very beginning of the second study (7^th^ of March, t-2), directly after closing the mesocosms but before mixing them with compressed air. pH_NBS_ profiles reveal that some of the high CO_2_ water from the first experiment was still present in some mesocosms (mainly M7 and M8) at the beginning of the second approach, even though mesocosm bags were completely under water and the sediment traps were removed during the 4 days in between the two studies.
