# Supplementary material for: Influence of Ocean Acidification on a Natural Winter-to-Summer Plankton Succession: First Insights from a Long-Term Mesocosm Study Draw Attention to Periods of Low Nutrient Concentrations
Source: PLoS One. 2016 Aug 15;11(8):e0159068. doi: 10.1371/journal.pone.0159068 (PMC4985126; doi:10.1371/journal.pone.0159068)
Supplement: S3 Fig — Changes in temperature averaged over the entire water column are represented by the white line plots on top of the contours with the corresponding y-axes on the right side. The black lines at t37 mark the end of convective mixing (See also Fig 5). (DOCX) [file pone.0159068.s003.docx]

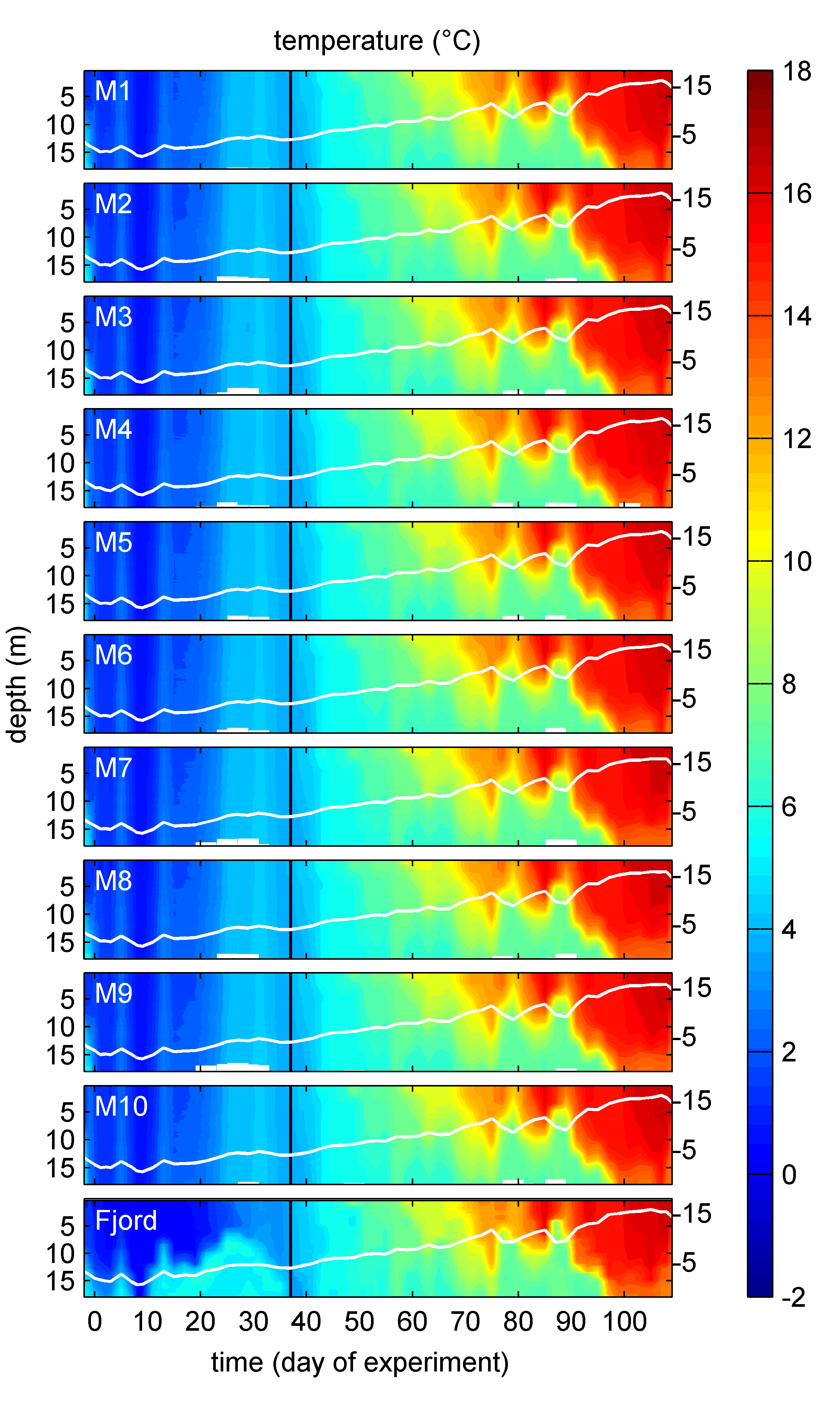


**S3 Fig.** Temperature profiles over the course of the study. Changes in temperature averaged over the entire water column are represented by the white line plots on top of the contours with the corresponding y-axes on the right side. The black lines at t37 mark the end of convective mixing (See also Fig 5).
