# Supplementary material for: Influence of Ocean Acidification on a Natural Winter-to-Summer Plankton Succession: First Insights from a Long-Term Mesocosm Study Draw Attention to Periods of Low Nutrient Concentrations
Source: PLoS One. 2016 Aug 15;11(8):e0159068. doi: 10.1371/journal.pone.0159068 (PMC4985126; doi:10.1371/journal.pone.0159068)
Supplement: S1 Table — Note that two studies [16,32] have already been published before initiating the PLOS collection. (DOCX) [file pone.0159068.s004.docx]

**S1 Table.** Contributions intended to be published within the framework of the BIOACID II long-term mesocosm study. Note that two studies [16,32] have already been published before initiating the PLOS collection.

| **Preliminary title of the paper** | **Key words** | **Lead author** | **PLOS or another journal?** |
| --- | --- | --- | --- |
| The influence of ocean acidification on the formation of naturally produced halocarbons | Ocean acidification, halocarbons, phytoplankton, KOSMOS | Abrahamsson, K. | PLOS |
| Ocean Acidification effects on mesozooplankton community development: results from a long-term near-natural conditions experiment | mesozooplankton community, copepods, hydromedusae, *Pseudocalanus* sp. | Algueró-Muñiz, M. | PLOS |
| Ocean acidification shows no detectable effect on swimming activity and body size in a common copepod | Climate change, pH decrease, zooplankton, copepods | Almén, A-K | PLOS |
| Influence of ocean acidification on phytoplankton succession in a coastal ecosystem | phytoplankton succession, picoeukaryotes, Nanophytoplankton, *Coscinodiscus* sp., autotroph to heterotroph ratio, | Bach, L. T. | PLOS |
| Abundance of the iron containing biomolecule, heme b, during the progression of a spring phytoplankton bloom in a mesocosm experiment. | hemoprotein, iron, KOSMOS, ocean acidification, pCO_2_, protoporphyrin (IX) | Bellworthy, J. | PLOS |
| Dynamics of carbon and nutrients during a natural winter-to-summer plankton succession at ambient and future CO2 concentrations | Element budget, mass balance, plankton succession, spring bloom, pelagic mesocosms, carbon dioxide, ocean acidification | Boxhammer, T. | PLOS |
| Vertical flux of marine particles during a natural winter-to-summer plankton succession in a mid-latitude coastal ecosystem. | Vertical flux, sinking velocity, stoichiometry, plankton community structure | Boxhammer, T. | PLOS |
| Elevated PCO_2_ and temperature affect survival, respiratory performance, and resource allocation during embryonic development of Atlantic cod *Gadus morhua* |  | Dahlke, F. | another journal |
| Mechanististic physiological model based on laboratory-based experiments successfully predicts sea urchin larval response to ocean acidification in mesocosms | Larval stages, sea urchins, physiology, model validation | Dupont, S, | PLOS |
| Impacts of ocean acidification on primary production in a coastal North Sea phytoplankton community | Ocean acidification, phytoplankton community | Eberlein, T. | PLOS |
| Application of stable carbon isotopes on a long term mesocosm study for carbon cycle investigation | carbon cycle, stable carbon isotope, phytoplankton, KOSMOS | Esposito, M. | PLOS |
| Low CO2 sensitivity of microzooplankton communities in the Gullmar Fjord, Skagerrak: evidence from a long-term mesocosm study | Ocean acidification, protozooplankton, ciliates, dinoflagellates, phytoplankton, plankton interactions | Horn, H. G. | PLOS |
| Effects of CO_2_-enrichment on bacteria-phytoplankton interactions during a winter-to-summer phytoplankton succession | Ocean acidification, CO_2_ enrichment, Baltic Sea, KOSMOS mesocosm experiment, bacterial production, phytoplankton | Hornick, T. | PLOS |
| Marine plankton communities show robustness under future ocean acidification conditions: Community barcoding results from a long-term mesocosm study in the North Sea | CO_2_, community barcoding, metabarcoding, NGS, ocean acidification, plankton | Lange, J. A. F. | PLOS |
| The role of the mitochondrial Complex I and Complex II in the thermal sensitivity of the embryos of Atlantic herring |  | Leo, E. | not decided |
| Experimental evolution gone wild | *Skeletonema*, diatom evolution, in-situ mesocosms, carbon dioxide, ocean acidification, experimental evolution | Scheinin, M. | already published in Royal Society Interfaces |
| Indirect effects of ocean acidification via the food chain on growth and survival of herring larvae in a mesocosm experiment | Ocean acidification, herring larvae, predator prey | Sswat, M. | PLOS |
| Performance and survival of larval Atlantic herring, *Clupea harengus*, under the combined effects of elevated temperatures and ocean acidification | Ocean acidification, temperature, herring larvae, low food condition | Sswat, M. | PLOS |
| Structural and functional responses of marine bacterial communities to ocean acidification | Ocean acidification, *Vibrio*, phytoplankton coupling, spring bloom, mesocosm, cultures, quantitative PCR, Sweden, BIOACID, KOSMOS 2013 | Stenegren, M. | PLOS |
| Ocean Acidification effects on mesozooplankton community development: results from a long-term near-natural conditions experiment | Ocean acidification, bacterial community, DGGE, bacterial abundance, copper resistance, antibiotic resistance. | Svensson, M | PLOS |
| Characterization of size structure and food-web composition during a winter-to-summer plankton succession using a zooplankton imaging system | Plankton imaging, particle size spectrum, biomass composition, food web structure | Taucher, J. | PLOS |
| Effect of ocean acidification on nitrogen cycling in a mesocosm study | Ocean acidification, nitrate uptake, new production, DON, recycled production | Wannicke, N. | PLOS |
| Influence of ocean acidification on the occurance of pycotoxins in the Gullmar Fjord. | Ocean acidification, harmful algae, phycotoxins, domoic acid | Wohlrab, S. | PLOS |
| Eukaryotic nano- and picoplankton diversity in a mesocom experiment. | Ocean acidifiaction, nanoplankton picoplankton, plankton community composition, DNA barcoding | Wohlrab, S. | PLOS |
| Effects of ocean acidification on marine dissolved organic matter are not detectable over the succession of phytoplankton blooms | Dissolved organic matter biogeochemistry, plankton succession, ocean acidification | Zark, M. | already published in Science Advances |
| Linking microbes to molecules | DOM dynamics, FT-ICR-MS, bacterial community, structure-function relationships | Zark, M. | PLOS |
