# Supplementary material for: Influence of Ocean Acidification on a Natural Winter-to-Summer Plankton Succession: First Insights from a Long-Term Mesocosm Study Draw Attention to Periods of Low Nutrient Concentrations
Source: PLoS One. 2016 Aug 15;11(8):e0159068. doi: 10.1371/journal.pone.0159068 (PMC4985126; doi:10.1371/journal.pone.0159068)
Supplement: S3 Table — For the community-based analysis we generally included functional groups rather than species. For copepods, we only included Pseudocalanus sp. here since this species strongly dominated the copepod community both, numerically and in terms of biomass. (Note, however, that “nauplii” includes copepod nauplii from all species since they were not distinguished taxonomically). This data was used for ANOSIM/NMDS analysis (Fig 8C). (DOCX) [file pone.0159068.s006.docx]

**S3 Table.** Abundance of individual plankton groups during peak chl*a* concentrations in phase III. For the community-based analysis we generally included functional groups rather than species. For copepods, we only included *Pseudocalanus* sp*.* here since this species strongly dominated the copepod community both, numerically and in terms of biomass. (Note, however, that “nauplii” includes copepod nauplii from all species since they were not distinguished taxonomically). This data was used for ANOSIM/NMDS analysis (Fig 8C).

|  | low CO_2_ | | | | | | | high CO_2_ | | | | | | |  |
| --- | --- | --- | --- | --- | --- | --- | --- | --- | --- | --- | --- | --- | --- | --- | --- |
| parameter | M1 | M3 | M5 | M9 | M10 | mean low CO_2_ | S.D. low CO_2_ | M2 | M4 | M6 | M7 | M8 | mean high CO_2_ | S.D. high CO_2_ | SIMPER (%) |
| days of peak bloom | t53, t55, t57 | t45, t47, t51 | t51, t57, t59 | t49, t51, t53 | t55, t57, t59 |  |  | t49, t51, t57 | t53, t55, t57 | t49, t51, t57 | t53, t55, t57 | t51, t53, t55 |  |  |  |
| *Coscinodiscus* (cells L^-1^) | 326 | 340 | 166 | 232 | 288 | 270 | 72 | 274 | 404 | 378 | 309 | 281 | 329 | 58 | 7.94 |
| Pico (cells mL^-1^) | 25598 | 8959 | 57203 | 39190 | 24157 | 31021 | 18135 | 55414 | 53286 | 64270 | 44692 | 81831 | 59899 | 14100 | 9.4 |
| Nano (cells mL^-1^) | 43471 | 3806 | 20179 | 12407 | 42332 | 24439 | 17826 | 10413 | 41176 | 16193 | 36655 | 74016 | 35691 | 25095 | 7.65 |
| Crypto (cells mL^-1^) | 96 | 139 | 79 | 94 | 97 | 101 | 23 | 252 | 62 | 202 | 36 | 62 | 123 | 97 | 8.34 |
| Synecho (cells mL^-1^) | 3225 | 550 | 637 | 605 | 4382 | 1880 | 1804 | 630 | 728 | 861 | 407 | 272 | 580 | 239 | 7.3 |
| Bacteria (cells mL-1) | 2458899 | 2002754 | 2701034 | 2829063 | 2728058 | 2543961 | 331658 | 2002754 | 1706444 | 2392019 | 2136357 | 1820039 | 2011523 | 269340 | 11.69 |
| Virus like particles (ind mL^-1^) | 36976667 | 40331905 | 44008095 | 27969048 | 37880952 | 37433333 | 5950017 | 40080952 | 32978095 | 38426190 | 37207143 | 43698571 | 38478190 | 3927166 | 7.05 |
| *Pseudocalanus* (male) (ind m^-3^) | 2684 | 2222 | 1328 | 2771 | 3175 | 2436 | 706 | 2251 | 2771 | 4214 | 2078 | 1674 | 2597 | 985 | 6.84 |
| *Pseudocalanus* (female) (ind m^-3^) | 6003 | 5224 | 2867 | 5051 | 5772 | 4983 | 1245 | 4329 | 6061 | 7504 | 6234 | 4541 | 5734 | 1312 | 7.11 |
| *Pseud*. Copepodites (ind m^-3^) | 50178 | 16662 | 21953 | 27956 | 31881 | 29726 | 12817 | 27860 | 66224 | 32189 | 44695 | 30438 | 40281 | 15884 | 7.53 |
| Nauplii (ind m^-3^) | 77884 | 60087 | 72862 | 96546 | 80635 | 77603 | 13204 | 107571 | 109110 | 115094 | 97278 | 80241 | 101859 | 13681 | 10.59 |
| Ciliates (cells mL^-1^) | 16840 | 9720 | 4320 | 2100 | 11400 | 8876 | 5856 | 1240 | 2900 | 2160 | 6340 | 9280 | 4384 | 3348 | 8.54 |
